# p53 Related Protein Kinase is Required for Arp2/3-Dependent Actin Dynamics of Hemocytes in *Drosophila melanogaster*

**DOI:** 10.3389/fcell.2022.859105

**Published:** 2022-06-02

**Authors:** Emiliano Molina, Vicente F. Cataldo, Cristián Eggers, Valentina Muñoz-Madrid, Álvaro Glavic

**Affiliations:** ^1^ FONDAP Center for Genome Regulation, Departamento de Biología, Facultad de Ciencias, Universidad de Chile, Santiago, Chile; ^2^ Department of Chemical and Bioprocess Engineering, School of Engineering, Pontificia Universidad Católica de Chile, Santiago, Chile; ^3^ Department for Chemistry and Biochemistry and Pharmaceutical Sciences, Faculty of Science, University of Bern, Bern, Switzerland

**Keywords:** cell migration, actin dynamics, PRPK, ARP2/3, Rab35, immune response

## Abstract

Cells extend membrane protrusions like lamellipodia and filopodia from the leading edge to sense, to move and to form new contacts. The Arp2/3 complex sustains lamellipodia formation, and in conjunction with the actomyosin contractile system, provides mechanical strength to the cell. *Drosophila* p53-related protein kinase (Prpk), a Tsc5p ortholog, has been described as essential for cell growth and proliferation. In addition, Prpk interacts with proteins associated to actin filament dynamics such as α-spectrin and the Arp2/3 complex subunit Arpc4. Here, we investigated the role of Prpk in cell shape changes, specifically regarding actin filament dynamics and membrane protrusion formation. We found that reductions in Prpk alter cell shape and the structure of lamellipodia, mimicking the phenotypes evoked by Arp2/3 complex deficiencies. Prpk co-localize and co-immunoprecipitates with the Arp2/3 complex subunit Arpc1 and with the small GTPase Rab35. Importantly, expression of Rab35, known by its ability to recruit upstream regulators of the Arp2/3 complex, could rescue the Prpk knockdown phenotypes. Finally, we evaluated the requirement of Prpk in different developmental contexts, where it was shown to be essential for correct Arp2/3 complex distribution and actin dynamics required for hemocytes migration, recruitment, and phagocytosis during immune response.

## Introduction

Cell migration is essential for morphogenetic movements and the recruitment of immune cells to infection sites (Ridley et al., 2003). Migratory cells extend and retract their plasma membrane depending on the forces that exert the underlying actin filaments (Callan-Jones and Voituriez, 2016). These membrane protrusions are classified according to their dynamics, structure, and proteins involved (Abella et al., 2016), the best characterized are lamellipodia and filopodia.

Lamellipodia are sheet-like structures that have a highly branched network of thin and short actin filaments. The lamella, a less branched and more linear actin arrangement, is associated and localized behind the lamellipodium (Cramer et al., 1997; Svitkina and Borisy, 1999). Lamellipodia are responsible for the generation of new contacts of the cell with the substrate, and together with myosin-II support cell motility in a 2D context (Vicente-Manzanares et al., 2009; Burnette et al., 2014; Leithner et al., 2016). Many extracellular stimuli such as growth factors and adhesion receptors induce lamellipodia by stimulating the small GTPase Rac1. Rac1-GTP stimulates the nucleation of branched actin by activating WAVE proteins (WAVE1-3, SCAR in *Drosophila*), which in turn activate the Arp2/3 complex (Ridley, 2011). Various groups have reported that cellular migration in confined contexts occurs even in the absence of lamellipodia formation machinery (Leithner et al., 2016; Linder, 2017; Rotty et al., 2017).

Filopodia are highly dynamic, long and thin protrusions of the cell formed by arrangements of parallel actin filaments that are stabilized by Fascin. Numerous mechanisms for the formation of filopodia have been described (Mattila and Lappalainen, 2008). In *Drosophila*, Cdc42 has been proposed to be exclusively involved in filopodia formation (Biyasheva et al., 2004). The Rab35 GTPase is a potent inducer of filopodia in S2 cells due to its ability to interact and recruit Fascin to the plasma membrane (Zhang et al., 2009). Also, Rab35 modulates the formation of lamellipodia in these cells by transferring Rac1 to the plasma membrane (Shim et al., 2010). In mammalian cells, Rab35 induces neurite outgrowth (Chevallier et al., 2009), suggesting a conserved function of this protein in regulating actin cytoskeleton and membrane protrusion dynamics.

p53-related protein kinase (PRPK) was reported in human activated T cells, showing its ability to phosphorylate p53 and to increase its transcriptional activity (Abe et al., 2001, 2006). However, its homologue in yeast, Bud32, was described as a small atypical acidic Ser/Thr kinase essential for cell growth (Stocchetto et al., 1997), and together with the RIO kinases are the only kinases conserved across eukaryotes and archea (Kannan et al., 2007; Kennelly, 2014; Beenstock and Sicheri, 2021). The relevance of Prpk and his homologues in cell growth is based on their integral role in the threonyl-carbamoyl transferase complex (TCTC), implicated in the N6-threonylcarbamoyl modification (t^6^A) of tRNAs. This modification is essential for cell viability, and mutants for the subunits of this complex exhibit cell growth and protein synthesis defects (El Yacoubi et al., 2011; Srinivasan et al., 2011; Ibar et al., 2013; Rojas-Benítez et al., 2013; Lin et al., 2015; Rojas-Benitez et al., 2015; Thiaville et al., 2016; Li et al., 2021).

Interestingly, despite the role of Prpk in tRNA modification, evidence shows it regulates cytoskeleton dynamics. In yeast, Bud32 mutants have defects in budding site selection (Kato et al., 2011), a process dependent on actin cytoskeleton dynamics (Casamayor and Snyder, 2002). Recently, studying the Galloway-Mowat syndrome (GAMOS), a condition associated with mutations in the TCTC complex subunits and characterized by microcephaly and early onset nephrotic syndrome (SRNS) (Braun et al., 2017; Edvardson et al., 2017), Braun et al. described that PRPK knockdown impairs actin dynamics and human podocyte migration (Braun et al., 2017). The function of PRPK regulating cytoskeleton dynamics could be explained by its interaction with proteins different from the TCTC complex subunits, such as Rab35, which has been shown to interact and regulate the cellular distribution of PRPK in human cells (Abe et al., 2006). Also, in mice Prpk negatively regulates axonal elongation by reducing the levels of Rab35 (Villarroel-Campos et al., 2016). Finally, pulldown assays coupled with mass spectrometry revealed the interaction of Prpk with α-spectrin and with the Arp2/3 subunit, Arpc4 in *Drosophila* (Guruharsha et al., 2011).

The interactions of Prpk with proteins that regulate actin cytoskeleton, prompted us to investigate its potential conserved function in actin cytoskeleton dynamics in migratory immune cells. Specifically stressing its functional interactions with Rab35, the small GTPases Cdc42 and Rac1, and their consequences for the formation of the parallel and the branched actin arrangements that sustain the dynamic behavior of membrane protrusions. To this end, we decided to evaluate Prpk function in *Drosophila* hemocytes, a differentiated and motile cell implicated in immune responses (Lemaitre and Hoffmann, 2007).

## Materials and Methods

### *Drosophila melanogaster* Strains

The following *Drosophila* strains were obtained from the Bloomington stock center: Cg-Gal4, Hml-Gal4, Hml^Δ^-Gal4, 2xEGFP, UAS-Rac1, UAS-Rac1^N17^, UAS-Cdc42^V12^, UAS-Cdc42^N17^, UAS-Arpc1-GFP, UAS-Rab35-YFP, UAS-Rab35^Q67L^-YFP, UAS-Rab35^S22N^-YFP, UAS-Xbp1-GFP, UAS-Rho^DN^, UAS-Rho1-GFP, UAS-mCD8:GFP, UAS-LifeAct:GFP, Nubbin-Gal4, En-Gal4, UAS-Cas9.P2, {TI}Rab35:EYFP and from the Vienna *Drosophila* Research Center: UAS-Arpc1-IR, UAS-Arpc4-IR, , sgRNA against dPrpk and sgRNA against white and UAS-Tor-IR. We used the same UAS-Prpk-IR and UAS-Prpk-FLAG strains as previously described (Ibar et al., 2013). We selectively performed gain or loss of function of Prpk and other proteins involved in cytoskeleton dynamics in hemocytes using the UAS/Gal4 system (Brand and Perrimon, 1993). All crosses were made at 25°C.

### p53-Related Protein Kinase Constructs

Prpk-HA N-terminal fusion was constructed amplifying the coding sequence from genomic DNA using the primers: 5′-CAC​CAT​GTC​CCT​AGA​AA-TCC​TGA​AAC​AAG​G-3′ and 5′-TTA​ACC​AAT​CAT​GGT​TCT​TTT​GCG​TCC-3´. The amplicon was cloned into the p-ENTR/D-TOPO vector (Invitrogen). Afterwards it was subcloned into the pTHW vector through Gateway technology (Invitrogen). A standard germ cell transformation was followed to obtain at least three independent transgenic insertions for each construct (Spradling and Rubin, 1982).

Wild Type and kinase-dead version of mice PRPK (PRPK and PRPK^KD^) were facilitated by the PhD González-Billault group (Villarroel-Campos et al., 2016). The coding sequences were amplified with the Q5 polymerase (thermofisher) using a forward primer that includes EcoRI cleavage site and a reverse primer that includes a NotI cleavage site.

Fw 5′ GAA​TTC​ACC​ATG​GCT​GGT​GTG​TCC​TCG​GAG​GCG 3’

Rv 5′ GCG​GCC​GCC​TAC​CCG​ACC​ATG​GAC​CGC​TTT​CGC 3’

The amplified fragments were then cloned into the pUAS attB vector, purified and subsequently injected into attP2 strain via the φC31 integrase system by BestGene Inc.

### Hemocytes Primary Cultures and Immunofluorescence

To prepare each primary culture coverslip, four third instar larvae were taken from the growing media and washed in PBS, until they were visibly free of food traces. They were subsequently passed through 70% ethanol for approximately 3 s to sanitize and washed once with PBS. In parallel, circular 12 mm coverslips were set by flaming them with absolute ethanol flaming and placing them on parafilm-coated slides. Larvae were placed on coverslips with 120 µl Schneider’s Insect Medium (Sigma-Aldrich); a small incision was made ​​in the posterior section of the cuticle (without damaging the fat tissue) immediately after under a dissecting scope and with dissecting forceps. The hemolymph was allowed to flow for 1 min; larvae were discarded afterwards. Hemocytes were allowed to adhere for 75 min at 25°C in a humidity chamber. After this time, coverslips were moved to a 12-well plate, medium was removed, and washed with PBS.

F-actin staining and immunofluorescence, cells were fixed in 4% PFA for 10 min, permeabilized with PBS−0.1% Triton for 10 min and blocked in PBS-BSA 1% for 1 h. The following primary antibodies were incubated overnight at 4°C: mouse monoclonal anti-FLAG (1:300, Sigma), rabbit polyclonal anti-FLAG (1:200, Sigma), mouse polyclonal anti-HA (1:200, Sigma), mouse monoclonal anti-GFP (1:200, Invitrogen), rabbit polyclonal anti-Arp2 (1:100, Abcam), mouse monoclonal anti-β-tubulin (1:10, Hybridoma Bank), and mouse monoclonal anti-Fascin (1:5, Hybridoma Bank. Alexa Fluor secondary antibodies (1:200, Invitrogen) and Phalloidin-FITC (50 μM, Sigma) were incubated for 2 h at room temperature, TO-PRO-3 (10 μM, Invitrogen) was added during the last 20 min of incubation with secondary antibodies. Incubation with Phalloidin/TO-PRO-3 and Phalloidin/DAPI (4 μM, Sigma) lasted for 1 h.

Imaging of cells was conducted on a Zeiss LSM510 confocal microscope with ×100 objective and 2.5 digital zoom, and/or a Zeiss Axiovert 200 M fluorescence microscope with ×100 objective.

### Cell Morphology Analysis

To distinguish the different phenotypes observed in primary culture hemocytes we manually recorded cell area, perimeter, area/perimeter ratio and number of protrusions from Phalloidin staining using a digitizing tablet (Wacom Intuos) and Image J. Both the area and the perimeter were quantified by segmenting the cells taking as reference the phalloidin staining. We consider as protrusion any portion of the actin stain that extends in front of the lamella and that adopts the morphology of a filament. The experiments were carried out in triplicate using four larvae to perform a primary culture and then at least 50 cells were quantified in each experiment, obtaining a total of 150 cells per condition.

### Co-Immunoprecipitation Assay

Prpk-HA and Arpc1-GFP constructs were expressed in fat bodies using the Cg-Gal4 driver. Then, 60 pairs of fat bodies were dissected from third instar larvae. We followed the protocol described Kim et al., 2002 for protein extraction with minor modifications. The RIPA buffer was replaced by a fat body extraction buffer (40 mM HEPES, pH 7.5, 120 mM NaCl, 10 mM Pyrophosphate, 10 mM Glycerophosphate, 50 mM NaF; 1.5 mM Na3VO4, 1 mM EDTA, 0.3% CHAPS and a cocktail of protease inhibitors) (Kim et al., 2002). Furthermore, we used the commercial kit GFP-Trap_A^®^ (Chromotek) immunoprecipitation. 25 μl of GFP-Trap_A^®^ beads were washed with 1 ml of dilution buffer (10 mM Tris-HCl pH 7.5; 150 mM NaCl; 0.5 mM EDTA and 1X of protease inhibitor mixture) three times. Beads were centrifuged at 2700 g for 2 minutes at 4°C during each wash, mixed with 250 μg of protein extract, and incubated for 2 h at 4°C with agitation. They were then washed twice with 500 μL of dilution buffer, with centrifugation at 2700 *g* for 2 minutes at 4°C between each wash. The supernatant was saved for posterior analysis and the pellet was re-suspended with 100 μl of 2X loading buffer, incubated at 65°C for 30 min, and centrifuged at 2700 g; the resulting supernatant was resolved in a 12% SDS-PAGE gel acrylamide-bisacrylamide (30%). Prpk-HA was revealed using an antibody against the HA epitope (1:500, mouse polyclonal, Santa Cruz) and GFP (1:1000, rabbit polyclonal, Santa Cruz). The images were processed using the UVITEC Alliance 4.7 system.

### *In vitro* Phagocytosis Assay

For phagocytosis assays, adherent hemocytes were incubated in a suspension of DH5α *E. coli* strain (2 × 10^7^ bacteria/ml) in Schneider’s Insect Medium for 15 min at 25°C. After washing the cells three times with cold PBS, they were fixed and stained with Phalloidin/TO-PRO-3 or Phalloidin/DAPI as previously mentioned. Hemocytes were fixed and stained after phagocytosis assays. F-actin was stained with Phalloidin and DNA with TO-PRO. The presence of internalized *E.coli* bacteria is evidenced by small rod-shaped TO-PRO staining, clearly different from the large and round shaped nucleus in hemocytes. The quantification was performed in Z-Stack Projections of hemocytes by counting the number of internalized bacteria per hemocyte and percentage of hemocytes with internalized bacteria.

### Hemocytes Recruitment Assay

Recruitment assays were conducted as described by Kadandale et al., 2010. Briefly, third instar larvae expressing EGFP under the Hml^Δ^Gal4 driver were damaged at the dorsal face between the A5 and A7 segments using a sharpened tungsten filament. The injury was performed avoiding the location of sessile hemocytes or crowded regions. Larvae were then transferred to agar-apple juice plates; after 6 h, the number of hemocytes recruited to the injury site were quantified and evaluated. For each condition (Control and Prpk-IR), four larvae were wounded and recorded. The experiment was done in quadruplicate. Hemocyte images were taken with an Olympus MVX10 fluorescence microscope, with ×1 objective and ×5 magnification.

### *In vivo* Migration Assay

Pupae between 15–20 h after puparium formation (APF) were mounted as described in (Moreira et al., 2011). Briefly, operculum was removed using tweezers, and pupae mounted with the ventral side down. A small amount of halocarbon was added to cover it and the migration of GFP-labeled hemocytes was recorded using a Carl Zeiss LSM710 confocal microscope with a ×40 objective. Videos are Z-projections of 15 slices of 1 μm each, acquired every 2 min during a 30-min period. The trajectory of the hemocytes was recorded using the ImageJ “Manual Tracking” plugin; their speed was measured using the “Chemotaxis Tool” plugin. n = 4 was used where at least seven hemocytes were quantified per animal.

### Live-Cell Imaging of Primary Culture Hemocytes

Primary hemocytes cultures and the subsequent analysis were performed as described in (Urra et al., 2018). Briefly, the LifeAct-GFP reporter was used to record actin dynamics and was expressed in hemocytes using the Cg-Gal4 driver. After 75 min of culture, films were made every 8 s for about 12 min using a Carl Zeiss LSM710 confocal microscope with a ×100 objective for each condition. Individual hemocytes were recorded in the plane where the largest membrane extension was observed. Velocity maps were generated using the ADAPT tool for ImageJ as indicated by the authors (Barry et al., 2015).

### Survival Rate Assay

Third instar larvae were pricked with a sharpened tungsten needle, previously dipped into a concentrated *E. coli* culture; 20 larvae were pricked for each condition. Pricked larvae were put on agar-apple juice plates for 6 hours. Larvae were then transferred to vials with fresh food and maintained at 25°C. After 7 days of incubation, the survival rate was measured by counting the adult flies present in each vial. The experiment was done in quadruplicate.

### Statistical Analysis

All the experiments were performed at least three times (indicated in the legends of the figures). Statistical analyses were performed using the “R” software environment (3.6.4 version). Morphological parameters were evaluated by Kruskal–Wallis followed with pairwise comparisons using Wilcoxon rank sum test, comparing all conditions. To analyze protrusions data, we compared the distribution using a Kolmogorov-Smirnov test. For all the conditions, we considered a Bonferroni correction.

For the hemocytes recruitment assay, Kruskal–Wallis tests were performed; differences were considered significant at *p* < 0.05 with n = 4 (four larvae per condition in each experiment). For the *in vivo* migration assay, two-tailed Paired *t*-Test were performed; differences were considered significant at *p* < 0.05 with n = 3 (quantified ≥6 hemocytes per pupa).

## Results

### Prpk Knockdown Alters Hemocytes Cell Shape

To investigate the participation of Prpk in actin cytoskeleton dynamics we decided to use *Drosophila* hemocytes based on the robustness of this model and the possibility of evaluating different immune contexts with high dependence on actin cytoskeleton dynamic (Wang et al., 2014; Vlisidou and Wood, 2015). All manipulation in protein levels in hemocytes were performed using the Gal4/UAS system for experiments *ex-vivo* or *in-vivo* (Brand and Perrimon, 1993) and CRISPR/Cas9 strategy (Port et al., 2020).

**FIGURE 1:**
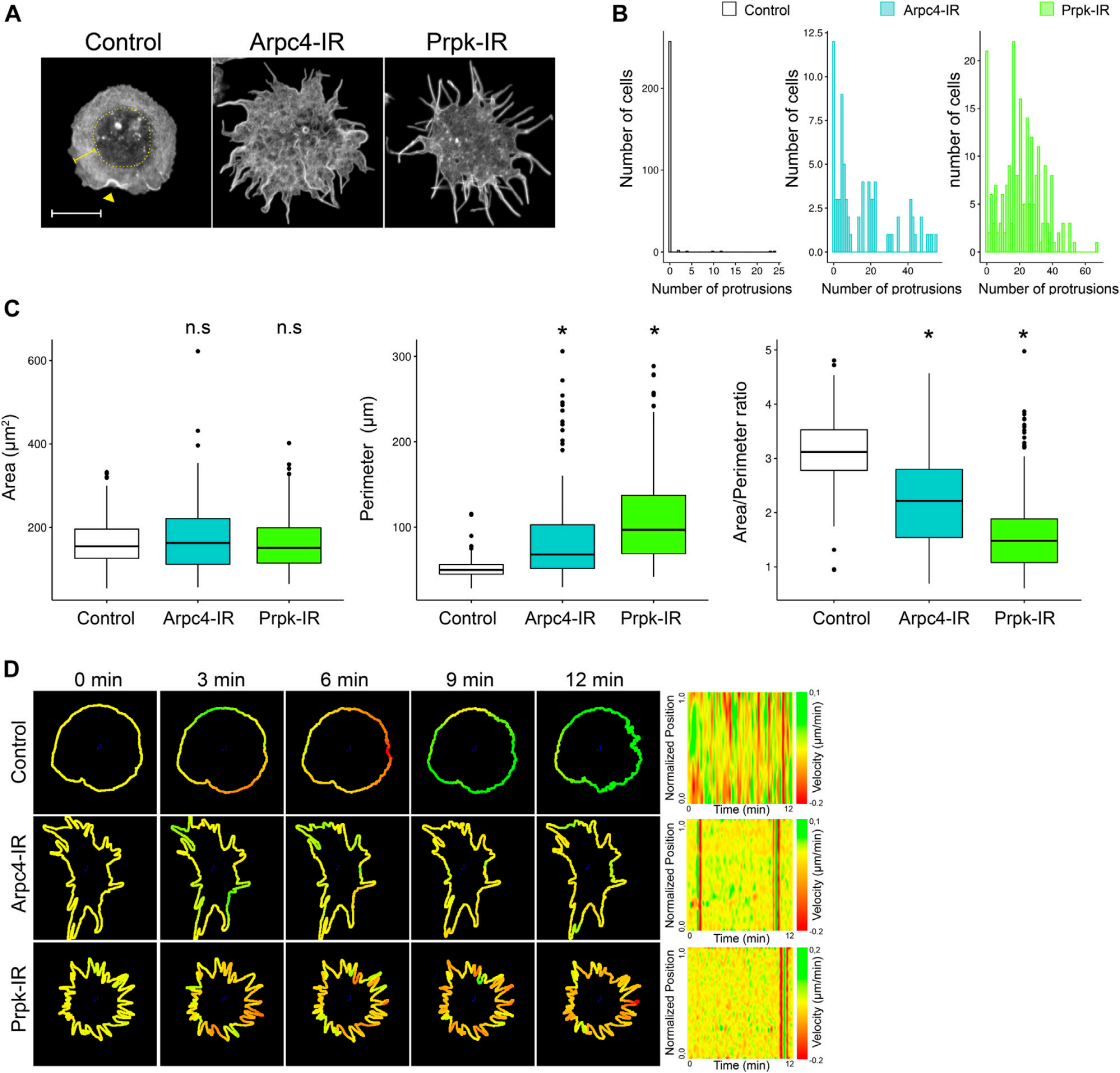
Knockdown of Prpk alters cellular shape of hemocytes, similar to loss of function of the lamellipodia actin nucleator Arp2/3. **(A)** Confocal images of F-Actin stained with phalloidin in primary culture of hemocytes expressing: mCD8:GFP (Control), Arp2/3 p20 subunit RNAi (Arpc4-IR) and Prpk RNAi (Prpk-IR) using Cg-Gal4 driver in each condition. The cell body corresponds to the innermost region of the cell, enclosed in the dotted circle, the lamella is depicted with a square bracket and the lamellipodia is visualized by actin ruffles (arrowhead). Scale bar: 10 μm. **(B)** Quantification of the number of protrusions for each condition. Data are presented as frequency of cells with protrusions. The Kolmogorov-Smirnov test was used for data analysis with a Bonferroni correction. Prpk-IR and Arpc4-IR hemocytes display a significant difference in the number of protrusions in relation to control cells (*p* < 0.005); n ≥4 (number of coverslips analyzed, quantified more than 150 cells in each case). **(C)** Cell morphology quantification expressed like Area, perimeter and Area/perimeter ratio for each condition. Kruskal–Wallis test was used for data analysis, followed by a pairwise comparisons using Wilcoxon rank sum test. *p < 0.001; n ≥4 (number of coverslips analyzed, quantified more than 150 cells in each case) **(D)** Time-lapses of the actin cytoskeleton were performed co-expressing LifeAct-GFP with the different RNAi constructs using the Cg-Gal4 driver. Velocity maps (Normalized position vs. Time) of representative cells for each condition were generated using the ADAPT tool. Regions that span the membrane are assigned a green color, while regions of retraction of the membrane have a red color. The transition between green and red regions denotes the contractile activity of the plasma membrane.

We first evaluated the participation of Prpk in the establishment of cell morphology and the dynamics of the actin cytoskeleton in primary cultures of hemocytes from third larval stage. To do this, we carry out F-actin staining of hemocytes, where control hemocytes (Figure 1A, Supplementary Figure S1A) show the presence of the three different actin distributions described also in S2 cells (Rogers et al., 2003). The cell body contains the nucleus and all organelles and is characterized by the weakest phalloidin stain. In this region actin filaments are arranged in typical cortical strands. The cell body is encompassed by the lamella, composed of densely configured linear actin filaments where the contractile machinery assembles (Ponti et al., 2004; Burnette et al., 2014). Finally, to the outside of the lamella, ruffles exhibit strong phalloidin staining, indicating more compact and branched configuration of actin filaments (Borm et al., 2005). These regions were identifiable in most hemocytes (Supplementary Figure S1A).

In contrast to control hemocytes, Arpc4 knockdown hemocytes (Arp2/3 p20 subunit RNAi, hereinafter referred to as Arpc4-IR), exhibited abnormal membrane protrusions highly enriched in actin filaments and the blurring of the cell body and lamella interphase (Figure 1A; Supplementary Figure S1A). Alterations also evoked by Arp2/3 deficiencies in other cellular systems (Suraneni et al., 2012, 2015; Leithner et al., 2016). A similar phenotype was observed in Prpk knockdown hemocytes (Prpk RNAi, hereinafter referred to as Prpk-IR), which showed an altered shape, with multiple elongated actin protrusions (Figures 1A,B; Supplementary Figure S1A). To be able to compare the different phenotypes observed, we counted the number of protrusions per cell and measured the area, perimeter and area/perimeter ratio of each cell. Prpk-IR and Arpc4-IR hemocytes have several protrusions unlike control hemocytes, which have a small proportion of cells with protrusions. Also, we can observe a greater number of hemocytes that have between 20 and 40 protrusions in Prpk-IR conditions compared to Arpc4-IR (Figure 1B). These differences can also be evidenced in the area/perimeter ratio, where the Prpk-IR hemocytes have a significant decrease in this parameter in relation to the control and Arpc4-IR hemocytes. This is due to the increase in the perimeter of hemocytes without altering the area (Figure 1C). We can also observe that the protrusions of the Arpc4-IR hemocytes tend to be thicker at the base unlike the Prpk-IR hemocytes (Figure 1A).

Prpk overexpression produced no overt phenotype, although it rescued the Prpk knockdown phenotype. Importantly, the phenotype was also rescued by expressing murine PRPK (Supplementary Figure S1B), confirming the conservation of Prpk function in this context and the specificity of the knockdown phenotype. Importantly, expression of a specific sgRNA against *dPrpk* and the Cas9 endonuclease (Port et al., 2020) in hemocytes using the Hml^Δ^-Gal4 driver mimics the Prpk-IR cellular phenotype (Supplementary Figure S1C).

Next, we compared the general cell shape characteristics of Arpc4 and Prpk knockdown cells with cells where canonical filopodia were induced by expressing a constitutively active version of Cdc42 (Cdc42^V12^) (Biyasheva et al., 2004). In contrast to Arpc4 and Prpk deficient cells, hemocytes expressing Cdc42^V12^ had fewer protrusions that extend from the cell border without losing the integrity of the lamella (Supplementary Figure S1D). Further consideration of the similarity between the Prpk and Arp2/3 knockdown cellular phenotypes instigated us to compare them with the effects caused by impairing Rac1, the main regulator involved in lamellipodia formation. Expression of a dominant negative form of Rac1, Rac1^N17^, did not elicit comparable phenotypes (Supplementary Figure S1D).

To get insights about protrusion dynamics induced in Prpk and Arpc4 knockdown conditions, we recorded cells co-expressing each construct and Lifeact-GFP (Figure 1D). Quantitation of Lifeact-GFP fluorescence at the periphery of the cell using the ADAPT tool (Barry et al., 2015; Urra et al., 2018), allowed us to estimate the extension and retraction of membrane protrusions. Arpc4 and Prpk-deficient cells displayed highly static actin protrusions, which can be clearly appreciated in the velocity maps (Figure 1D; Supplementary Videos S1–S3). This contrasts to what are observed in control (Figure 1D; Supplementary Video S1) and Cdc42^V12^-expressing cells (Supplementary Figure S2A; Supplementary Video S4), which exhibited highly dynamic activity of protrusions in Cdc42^V12^ cells or extension and retraction in control cells. Overall, comparisons of protrusions dynamics suggest that extensions observed in Arpc4 and Prpk knockdown cells do not behave as canonical filopodia.

Tsc5p and Prpk have been described as essential elements for t^6^A modification of tRNAs and therefore for protein synthesis (Srinivasan et al., 2011; Ibar et al., 2013; Rojas-Benítez et al., 2013; Rojas-Benitez et al., 2015). We previously reported that knockdown of Tcs5 in the whole animal inhibits the PI3K/TOR signaling pathway, detected by the reduction in the levels of phosphorylation (activation) of its targets S6K and 4 EBP (Ibar et al., 2013). Hence, the hemocyte phenotype produced by Prpk depletion might be the result of deficient cell growth or cellular stress mediated by a downregulation of the PI3K/TOR pathway. To rule this out, hemocytes expressing an interfering RNA against TOR (TOR-IR) or a dominant-negative form of PI3K (PI3K^D954A^) were analyzed. PI3K^D954A^ cells exhibited normal morphology, while TOR knockdown cells were abnormal, although dissimilar to Prpk-deficient hemocytes (Supplementary Figure S3A). In addition, co-expression of Prpk-IR and the UPR reporter, Xbp1-GFP (Ryoo and Steller, 2007), displayed exclusively the basal fluorescent signal (Supplementary Figure S3B). These results indicate that hemocyte protrusions induced by Prpk knockdown do not depend on its role in protein synthesis or alteration of PI3K/TOR pathway and is more likely related with its potential function in actin cytoskeleton dynamics.

### Protrusions in Prpk-Knockdown Hemocytes Lack Fascin and Are Similar to Protrusions Observed in Cells With Reduced Lamellipodia

Leithner et al. (2016) described that the abundance of filopodia favors the response to chemotactic stimuli but reduces the migratory capacity in geometrically complex environments (Leithner et al., 2016). Filopodia have also been shown to act as molds for the formation and orientation of lamellipodia (Johnson et al., 2015).

**FIGURE 2:**
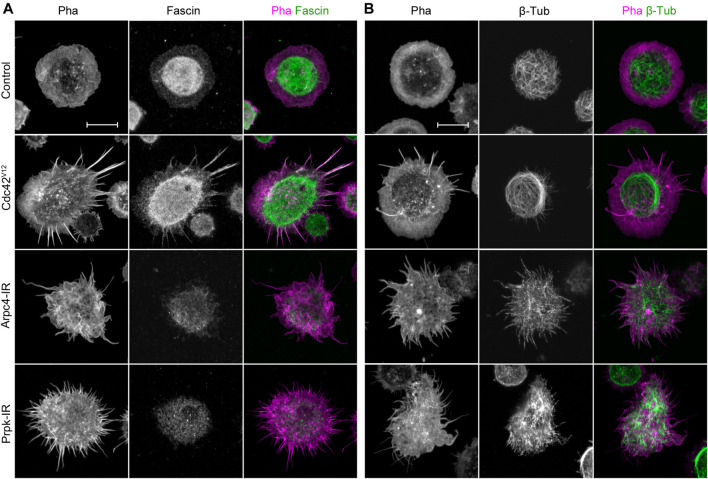
Membrane protrusions in Prpk knockdown hemocytes are not canonical filopodia but are similar to protrusions observed in loss of lamellipodia condition. **(A)** Immunostaining of primary culture of hemocytes for F-actin with Phalloidin (Pha, red) and filopodia marker Fascin (green). **(B)** Immunostaining for F-actin (Pha, magenta) and β-Tubulin (green). Expressions: Control (Cg-Gal4 driver), Arp2/3 p20 subunit RNAi (Arpc4-IR), Prpk RNAi (Prpk-IR) and constitutively active Cdc42 (Cdc42^V12^). Loss and gain of function conditions were induced in hemocytes using the Cg-Gal4 driver for each condition. Scale bars: 10 μm.

Our results on actin cytoskeleton dynamics suggest that protrusions produced by Prpk deficiency do not correspond to filopodia. Reports show that Fascin stabilizes parallel actin filaments in filopodia and can be used as a marker for this type of extensions (Kureishy et al., 2002; Suraneni et al., 2015). Therefore, and to confirm our observations, we compared the distribution of Fascin in Arpc4-IR, Prpk-IR, and Cdc42^V12^ hemocytes (Figure 2A). In control hemocytes, Fascin was detected at low levels mainly at the cell body region and to a lesser extent in the lamella. Protrusions in Arpc4-IR and Prpk-IR hemocytes exhibited no Fascin, indicating that these processes lack the classic filopodial composition. In contrast, in Cdc42^V12^ hemocytes Fascin was detected in the filopodia.

Another characteristic of filopodia is the absence of microtubules (Letourneau, 1983). Protrusions induced by silencing Arpc4 in S2 cells, however, do contain these cytoskeletal elements (Rogers et al., 2003). In control hemocytes, the microtubule network was located mainly in the cortex of the cell body (Figure 2B). Hemocytes depleted of Arpc4 had a series of protrusions with short microtubules. A similar distribution was observed in Prpk-IR cells; however, microtubules were present preferentially in the wider protrusions. Cells expressing Cdc42^V12^ showed an invasion of microtubules in the lamella, but as expected, these were not found in the protrusions. Together, these observations strengths the notion that membrane processes generated in Prpk deficient hemocytes are not canonical filopodia, and their characteristics are alike those induced by Arp2/3 complex inhibition.

### Prpk Interacts With Arpc1 and Influences the Distribution of Arp2/3 Subunits

**FIGURE 3:**
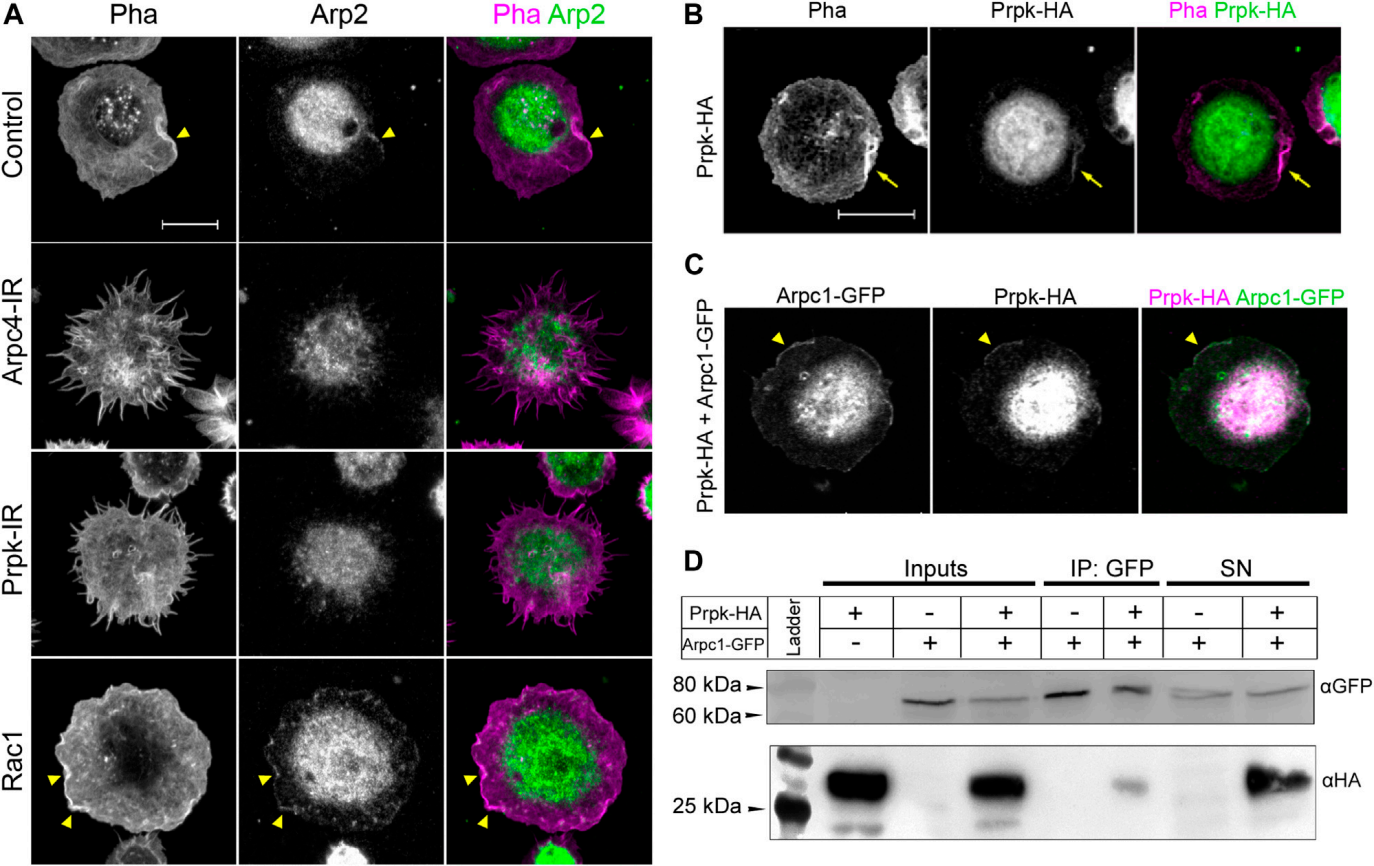
Prpk colocalize with Arp2/3 complex and physically interacts with Arpc1 subunit. **(A)** Immunofluorescence against Arp2 subunit (Arp2, green) of primary culture of hemocytes expressing: Cg-Gal4 driver (Control), Arp2/3 p20 subunit RNAi (Arpc4-IR), Prpk RNAi (Prpk-IR) and Rac1 GFPase (Rac1) using Cg-Gal4 driver in each condition. F-actin was stained with phalloidin (Pha, magenta). Arrowhead indicates lamellipodia-like ruffles. **(B)** Immunofluorescence of cultured hemocytes stained for F-Actin (Pha, magenta) and overexpressing Prpk-HA (green). **(C)** Immunofluorescence of hemocytes overexpressing Prpk-HA and Arpc1-GFP. Arrowhead indicates co-localization in lamellipodia-like ruffles **(D)** Co-immunoprecipitation between Prpk-HA and Arpc1-GFP. Inputs, Immunoprecipitation and Supernatants (SN) conditions are detailed in each case. The image corresponds to a representative blot, n = 2. Constructs expression was induced using the Cg-Gal4 driver for each condition. Scale bars: 10 μm.

Due to the similarity between Prpk-IR and Arpc4-IR phenotypes, we further investigated the distribution of two subunits of the Arp2/3 complex in the lamellipodia: Arp2 and Arpc1. Arp2 protein was enriched in ruffles (arrowhead in Figure 3A), but also distributed along the cell body and the lamella of control cells (Figure 3A). In accordance with its preferential distribution in the lamellipodia the accumulation of Arp2 in this structure was increased in hemocytes overexpressing Rac1 (Figure 3A, Rac1 panel). Conversely, Arpc4-IR and Prpk-IR hemocytes exhibited Arp2 localization in the cell body and in the protrusions. In addition, the distribution of Arpc1-GFP fusion protein was similar to that observed with Arp2 antibody (Supplementary Figure S4A). These results suggest that protrusions in Prpk-IR and Arpc4-IR hemocytes might result from the collapse of the lamellipodia and lamella.

Under the hypothesis that Prpk is necessary for formation and maintenance of the lamellipodia, we reasoned that Prpk could co-distribute with the Arp2/3 complex in this structure. Prpk-HA expressed in hemocytes distributed in the cell body and lamellipodia (arrow in Figure 3B); a weak expression of Prpk-HA was observed in the lamella. The co-expression of Prpk-HA and Arpc1-GFP showed that both proteins co-distributed in cell body and ruffles (arrowhead in Figure 3C), while the expression of EGFP was observed only in cell body and lamella (Supplementary Figure S4B). Finally, we evaluated the interaction between Prpk and the Arp2/3 complex via a co-immunoprecipitation assay. Two different tagged versions of Prpk (Prpk-HA and Prpk-Flag) were able to co-immunoprecipitate with Arpc1-GFP (Figure 3D; SupplementaryFigure S5). These results show that Prpk interacts with the Arp2/3 complex, regulating the location and perhaps the function of this complex and in consequence knocking down Prpk impairs lamellae and lamellipodia formation disturbing also Arp2/3 complex distribution.

### Prpk Interacts With Rab35 to Regulates Cell Shape and Lamellipodia Structuration

Since Prpk co-distributes with Arp2/3 subunits at the lamellipodia and Prpk-IR phenotypes were like those evoked by Arp2/3 deficiencies, we evaluated the functional interaction of Prpk with upstream activators of Arp2/3 complex. One of the most attractive candidates is Rab35, which localizes both at the plasma membrane and endosomes, and is involved in several process like endosomal trafficking, actin dynamic, phagocytosis, cell migration, neurite outgrowth among others (Klinkert and Echard, 2016). Importantly, it has been described its interaction with PRPK in human cells (Abe et al., 2006) and murine model (Villarroel-Campos et al., 2016). In addition, Rab35 has been shown to regulate the assembly of actin filaments in filopodia and lamellipodia in *Drosophila* (Shim et al., 2010; King et al., 2016).

**FIGURE 4:**
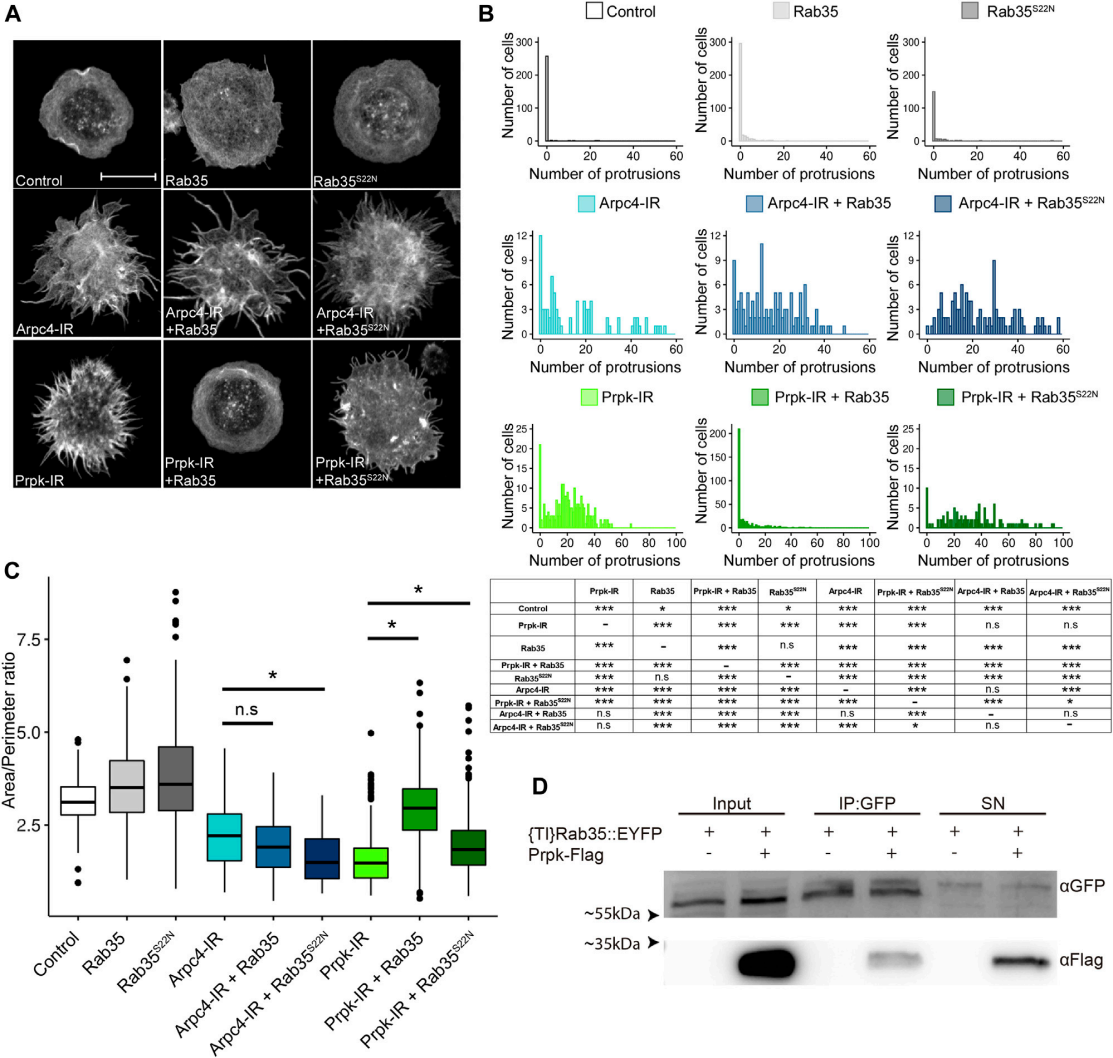
Prpk functionally interacts with Rab35 in cell shape maintenance and lamellipodia structuration. **(A)** F-Actin stain with Phalloidin in hemocytes. Loss and gain of functions were induced in hemocytes using the Cg-Gal4 driver for each condition: mCD8:GFP **(**Control), Rab35 tagged to YFP (Rab35), YFP tagged version of a dominant negative form of Rab35 (Rab35^S22N^), Arpc4-IR, Arpc4-IR + Rab35, Arpc4-IR + Rab35^S22N^, Prpk-IR, Prpk-IR + Rab35 and Prpk-IR + Rab35^S22N^. Scale bar: 10 μm. **(B)** Quantification of the number of protrusions for each condition. Data are presented as frequency of cells with protrusions and the statistical differences are summarized in the inserted table. The Kolmogorov-Smirnov test was used for data analysis, with a Bonferroni correction: *p < 0.05, **p < 0.01, ***p < 0.001; n ≥4 (number of coverslips analyzed, quantified more than 150 cells in each case). **(C)** Cell morphology quantification expressed like Area/perimeter ratio for each condition. Kruskal–Wallis test was used for data analysis followed with pairwise comparisons using Wilcoxon rank sum test. All the conditions evaluated show significant differences with the control. Statistical differences are indicated by bars. *p < 0.001; n ≥4 (number of coverslips analyzed, more than 150 cells were quantified in each case) **(D)** Co-immunoprecipitation between Prpk-Flag and {TI}Rab35:EYFP. Inputs, Immunoprecipitation and Supernatants (SN) conditions are detailed in each case. The image corresponds to a representative blot, n = 3.

We therefore decided to investigate their functional relations by co-expressing Prpk-IR with a form of Rab35 tagged to YFP (Rab35) and with its dominant negative form also tagged to YFP (Rab35^S22N^). The expression of Rab35 in hemocytes has been shown to generate a mild induction of filopodia, while Rab35^S22N^ expression in these cells results in a reduced formation of lamellae (Shim et al., 2010). The expression of Rab35 weakly induced filopodia in our system (Figures 4A,B), while the expression of Rab35^S22N^ produced a subtle augment in the area and area/perimeter ratio (Figure 4C; Supplementary Figure S6A), probably due to the reported cytokinesis defects in Rab35 deficient cells (Kouranti et al., 2006). Co-expression of Rab35-YFP with Prpk-IR partially reverse the Prpk knockdown phenotype, resulting in a significant reduction in the number of cells with protrusions, compared to Prpk-IR cells (Figures 4A,B). On the other hand, Rab35^S22N^ did not substantially modify Prpk-IR phenotype (Figures 4A,B). In contrast, co-expression of Arpc4-IR with Rab35, or Rab35^S22N^, did not modify the Arpc4-IR phenotype (Figures 4A,B). Interestingly, we also observed a significant increase in the number of protrusions of the Arpc4-IR and Prpk-IR hemocytes when Rab35^S22N^ was co-expressed, likely due to defects in lamellae formation (Shim et al., 2010). The reversal of the Prpk-IR phenotype can be also observed in the area/perimeter ratio, with a significant difference between Prpk-IR and co-expression of Prpk-IR and Rab35, while Rab35 did not exert any effect on the Arpc4-IR phenotype (Figure 4C; Supplementary Figure S6A).

The functional interactions between Prpk and Rab35 where further supported by the physical interaction detected between these proteins by co-immunoprecipitation of Prpk-Flag and the knock-in YFP fusion version of Rab35 ({TI}Rab35:EYFP) (Figure 4D). These results suggest that the role of Rab35 in actin cytoskeleton structuration requires the downstream activity of the Arp2/3-complex for the formation of the lamellipodia and the defects produced by reducing Prpk can be partially reverted by this protein suggesting that lamellipodia, which is dependent on Arp2/3 and Rac1, can be regulated by the activities of Prpk and Rab35 in these cells.

It has been reported that Rab35 is able to recruit the GTPases Rac1 and Cdc42 to the plasma membrane, which could explain its ability to rescue the phenotype by favoring the formation of lamellipodia (Shim et al., 2010). Rac1 is the main inducer of lamellipodia through the Arp2/3 complex, and while Cdc42 has been primarily described in filopodia formation (Ridley, 2011). In *Drosophila* hemocytes, Rac1 over-expression stimulates the formation of lamellipodia, revealed as ruffles (Williams et al., 2006). We also observed this in our model, but interestingly, co-expression of Prpk-IR and Rac1 attenuated the Prpk knockdown phenotype, resulting in a partial reduction of cells with protrusions (Supplementary Figures S6B,C). However, silencing Prpk also impaired the ability of Rac1 to induce ruffles formation (Supplementary Figure S6B). Thus, Prpk is required for the full lamellipodia inducing activity of Rac1 overexpression and therefore protrusions caused by the Prpk depletion may be related to defects in the formation of lamellipodia.

As expected Cdc42^V12^ expression induces filopodia (Ridley, 2011) (Supplementary Figures S6B,C) and the co-expression of Prpk-IR and Cdc42^V12^ exhibited no apparent formation of lamellae and cells were completely spherical, possibly due to their inability to spread over the substrate (Supplementary Figure S6B). Likewise, the co-expression of Cdc42^V12^ and Arpc4-IR also induced round cells with no lamellae (Supplementary Figure S6B), suggesting similar synthetic interactions between Cdc42 and both Prpk and Arp2/3 deficiencies.

### Migration, Recruitment, and Phagocytosis Are Affected in Prpk-Depleted Hemocytes

The Arp2/3 complex is responsible for the branched polymerization of actin, which is essential for cell migration. However, several studies have shown that cell migration can occur at the expense of the Arp2/3 complex, which reveals the redundancy of actin regulators, and the compensatory mechanisms present in cells (Suraneni et al., 2015; Leithner et al., 2016; Rotty et al., 2017). Nonetheless, Arp2/3 complex has been described to be crucial for haptotaxis (King et al., 2016). Remarkably, *Drosophila* allows the study of matrix dependent migration (haptotaxis) during the pupal development *in-vivo*. During this stage, hemocytes randomly migrate and interact through integrins with the extracellular matrix, which is decisive for their efficient migration (Moreira et al., 2011, 2013). In this context, it has been reported that the loss of function of both SCAR and the Arp2/3 complex promote the formation of protrusions, but these hemocytes migrate slower (Sander et al., 2013; Swaney and Li, 2016).

**FIGURE 5:**
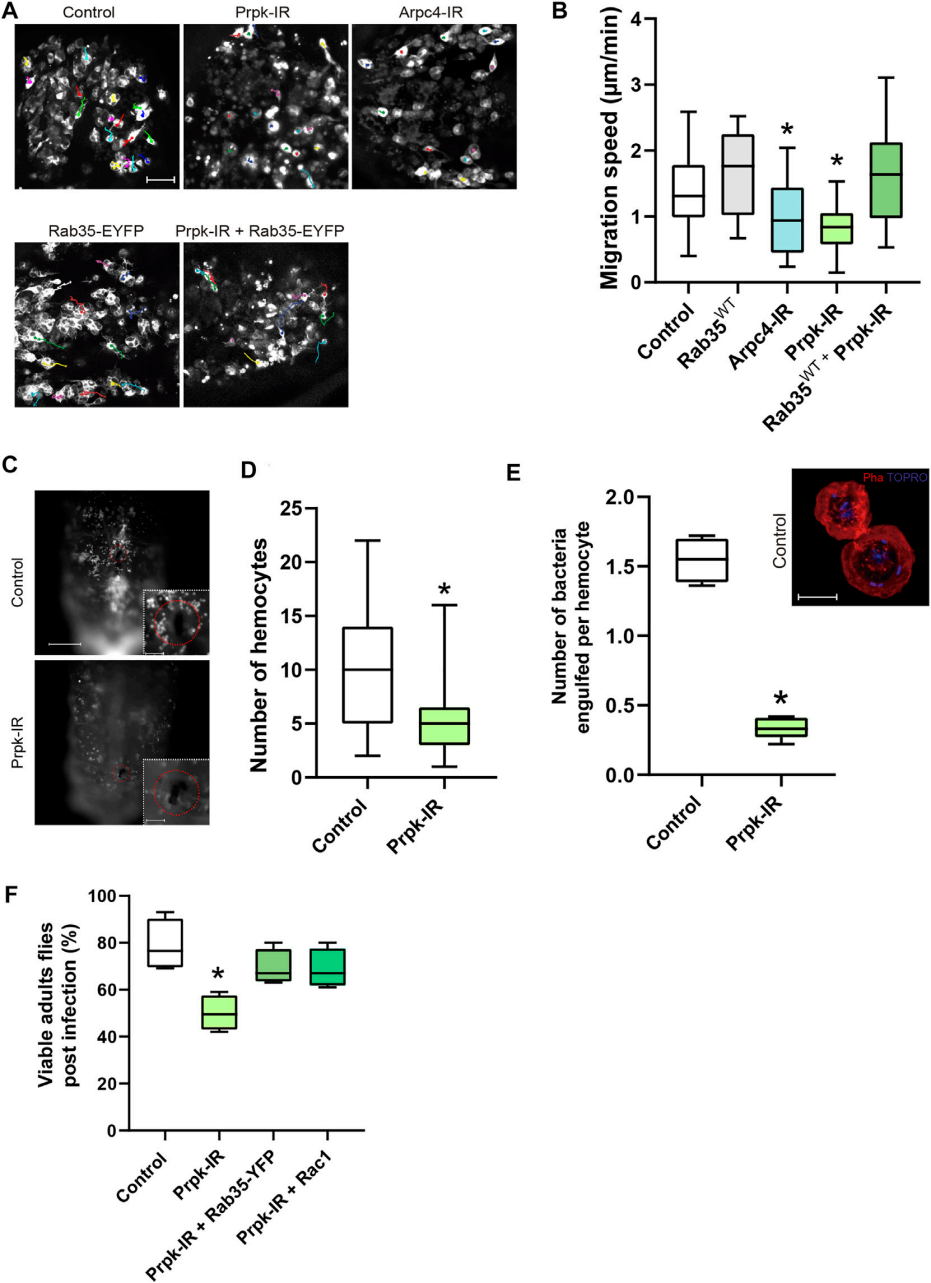
Prpk is essential for hemocyte recruitment, migration and phagocytosis during the immune response. **(A)** Time-lapse of Z-stack projections of pupal hemocytes of 15–20 h after puparium formation (APF) expressing GFP specifically in hemocytes with the Hml-Gal4 driver (Hml-Gal4, 2xEGFP): Hml-Gal4, 2xEGFP (Control), overexpression of Rab35^WT^-YFP (Rab35-EYFP), Prpk-IR, co-expression of Prpk-IR and Rab35^WT^-YFP and Arpc4-IR. **(B)** Quantification of migration speed for each condition. The trajectories of hemocytes were labeled by color tracks. * corresponds to *p* < 0.05 (Kruskal–Wallis test, n = 4, where at least seven hemocytes were quantified per animal). Scale bar: 50 μm. **(C)** Recruitment assay in response to tissue damage. Scale bar: 150 μm in full-images and 50 μm in zoomed images. **(D)** The number of hemocytes recruited to the wound site were quantified 6 h after damage in control and Prpk-IR larvae. * means *p* < 0.005 (Mann-Whitney two-tailed test, n = 3, 10 larvae per experiment). **(E)** Quantification of the number of bacteria engulfed per hemocytes. * corresponds to *p* < 0.01 (Mann-Whitney two-tailed test, n = 5). Z-Stack Projections of control hemocytes fixed and stained after phagocytosis assays. F-actin stained with Phalloidin (Pha, red), and DNA with TO-PRO (blue). The presence of internalized *E.coli* bacteria is evidenced by small rod-shaped TO-PRO staining. Scale bar: 10 μm. **(F)** Chart depicting the percentage of viable adult flies subjected to infection with *E.coli*.* correspond to *p* < 0.05 (Mann-Whitney two-tail test, n = 4, 20 flies per experiment).

In order to evaluate the requirement of Prpk in a matrix dependent migration context, we performed a knockdown of Prpk or Arpc4 in pupal hemocytes using the Hml^Δ^-Gal4, 2xEGFP driver and quantify the random velocity migration. Track of pupal hemocytes showed that Arpc4 and Prpk knockdown hemocytes migrate less and slower than control cells (Figures 5A,B; Supplementary Videos S5–9). Considering our previous results, we decided to evaluate whether the migratory defects produced by Prpk knockdown could be rescued by Rab35 overexpression *in-vivo*. Co-expression of Rab35-YFP and Prpk RNAi rescued the slower cell migration (Figures 5A,B) but these hemocytes mainly form filopodia (Supplementary Figure S7, Supplementary Video S10). These *in vivo* observations, in the context of random migration, further confirmed the functional interaction between Rab35 and Prpk.

At larval stages, *Drosophila* hemocytes are distributed in discrete anatomical regions. A sessile population clusters in gastrointestinal system and larval body wall, a second population resides in the lymph gland and proliferates in response to infections and finally most of the hemocytes are free in hemolymph and can be recruited in response to tissue damage (Evans and Wood, 2014; Gold and Brückner, 2015). We assessed cell recruitment and adhesion of hemocytes 6 hours after aseptically cuticle damage (Kadandale et al., 2010). Prpk-IR hemocytes were recruited to the wound site significantly less than control cells (Figures 5C,D).

As a part of the immune response hemocytes engulf pathogens in larval and adult stages (Lemaitre and Hoffmann, 2007), a process highly dependent on actin dynamics. We decided to assay bacterial phagocytosis in primary cultures of control and Prpk deficient cells. Larval Prpk-IR hemocytes exhibited a 49% reduction in the percentage of cells with internalized bacteria and a 78% reduction in the number of bacteria internalized per cell (Figure 5E). As previously mentioned, the Arp2/3 complex is essential for proper lamellae and lamellipodia formation, which are key structures for cell motility and phagocytosis (May et al., 2000; Insall et al., 2001; Sander et al., 2013; Rotty et al., 2017). Thus, the detrimental effect in phagocytosis observed in Prpk-IR hemocytes could be due to a diminished function of the Arp2/3 complex in these cells.

Finally, we wanted to evaluate the efficiency of the immune response after subjecting animals to an infection during the third larval stage. To do this, we performed the previously described assay of hemocytes recruitment and adhesion but in this case we used a tungsten needle previously immersed in an *E.coli* culture and we evaluated survival rate in the adult stage. In this assay we observed a lower survival rate in response to *E. coli* infection in animals with Prpk-deficient hemocytes (50%) than in control animals (>75%) (Figure 5F). This increase in mortality upon infection in Prpk-IR condition can be explained by the reduced recruitment (Figures 5C,D) and phagocytic capacity of Prpk-IR hemocytes (Figure 5E).

Since Rac1 and Rab35 were able to recover the cell shape and migration phenotypes produced by Prpk deficiency (Figures 4A–C; Supplementary Figures S6B,C), we also examined the rate of survival to infection in flies co-expressing each protein with the Prpk-IR construct. In line with our previous results, Rac1 and Rab35 were able to partially recover the survival rate up to 69% in each case (Figure 5F). Together, our results indicate that Prpk is essential for hemocytes to properly implement an immune response and that both, Rac1 and Rab35, not only revert the morphological and cytoskeletal defects produced by Prpk silencing but are also able to partially restore the functionality of immune cells. The results presented highlight the importance of Prpk in phagocytosis, migration and recruitment, processes fundamental for the proper execution of the immune response and the survival of the organism challenged by an infection.

### The Kinase Activity of Prpk Is Essential for the Determination of Cell Shape

**FIGURE 6:**
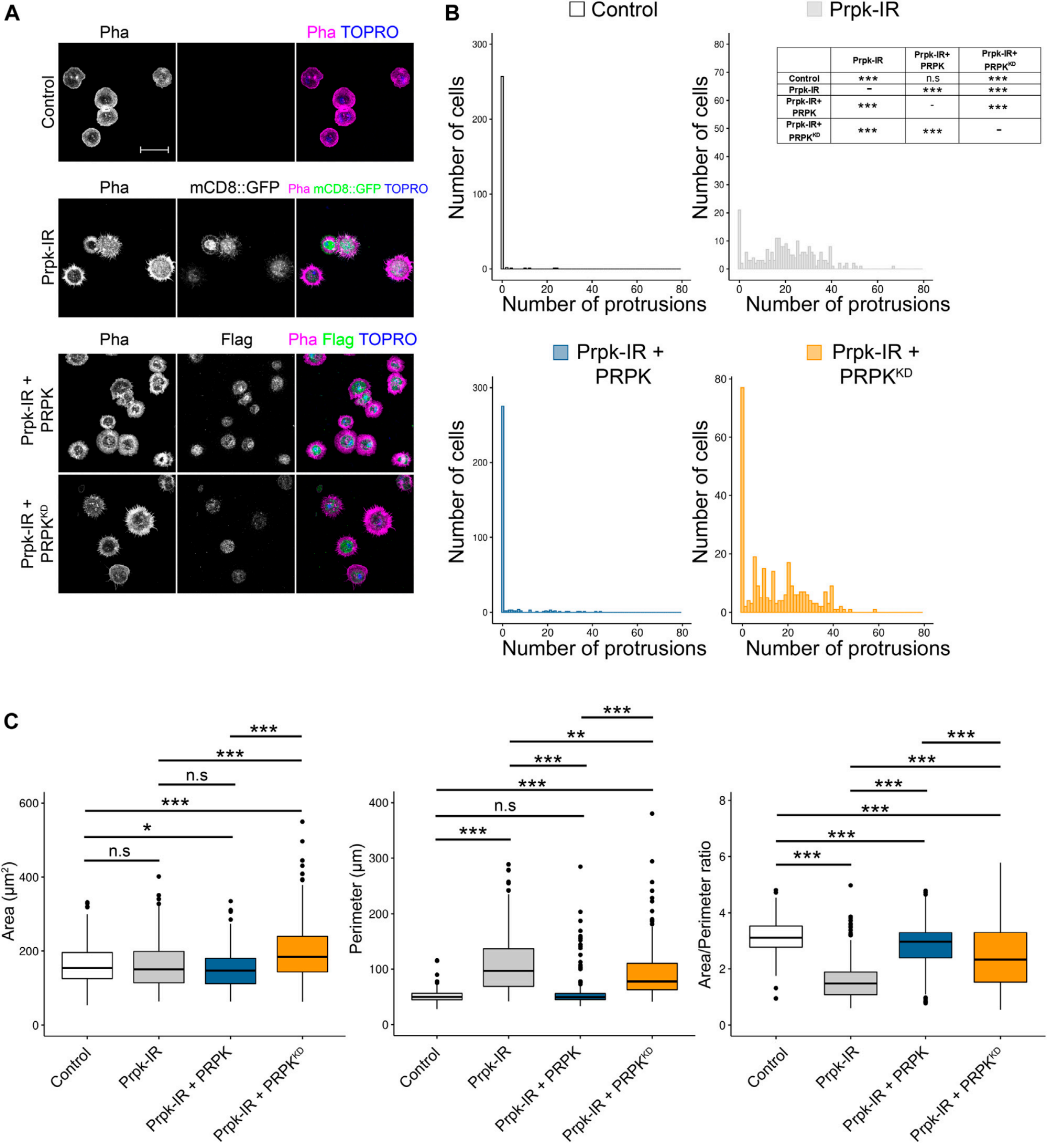
Prpk knockdown phenotype can be rescued by the co-expression of murine orthologue (PRPK) but not by the kinase dead version of PRPK (PRPK^KD^). **(A)** Confocal images of F-Actin stained with phalloidin in primary culture of hemocytes expressing: mCD8:GFP **(**Control), Prpk RNAi (Prpk-IR), Prpk-IR + PRPK and Prpk-IR + PRPK^KD^ using Cg-Gal4 driver in each condition. Scale bar: 10 μm. **(B)** Quantification of number of protrusions for each condition detailed previously. Data are shown as frequency of cells with protrusions and the statistical differences are summarized in the inserted table The Kolmogorov-Smirnov test was used for data analysis, with a Bonferroni correction: *p < 0.05, **p < 0.01, ***p < 0.001; n ≥ 4 (number of coverslips analyzed, quantified over 150 cells in each case). **(C)** Cell morphology quantification expressed like Area, perimeter and Area/perimeter ratio for each condition. Kruskal–Wallis test was used for data analysis, followed with pairwise comparisons using Wilcoxon rank sum test, comparing all conditions to each other. *p < 0.05, **p < 0.01, ***p < 0.001; n ≥ 4 (number of coverslips analyzed, quantified over 150 cells in each case).

It has been reported that Prpk has ATPase activity when it is part of the TCTC complex and kinase activity in a context outside the TCTC complex (Perrochia et al., 2013). Therefore, we wanted to assess the impact of kinase activity in determining hemocyte morphology. In order to this, we co-express the mouse homologue of Prpk (PRPK) or its kinase-dead version (Prpk^KD^) with the Prpk RNAi in primary cultures of hemocytes using the Cg-Gal4 driver. Importantly, mice PRPK was able to rescue the Prpk knockdown phenotype, with a significant reduction in the number of protrusions, while PRPK^KD^ was not able to rescue the Prpk-IR phenotype (Figure 6A). Further, PRPK could rescue morphological parameters like perimeter and Area/Perimeter ratio, while PRPK^KD^ only partially rescued these parameters (Figures 6B,C). This suggests that the role of Prpk associated with the dynamics of the actin cytoskeleton is conserved and very likely related with its kinase activity. However, understand the precise mechanism requires further investigations.

### Prpk Knockdown Strongly Reduces Myosin Light Chain II Phosphorylation in Hemocytes

**FIGURE 7:**
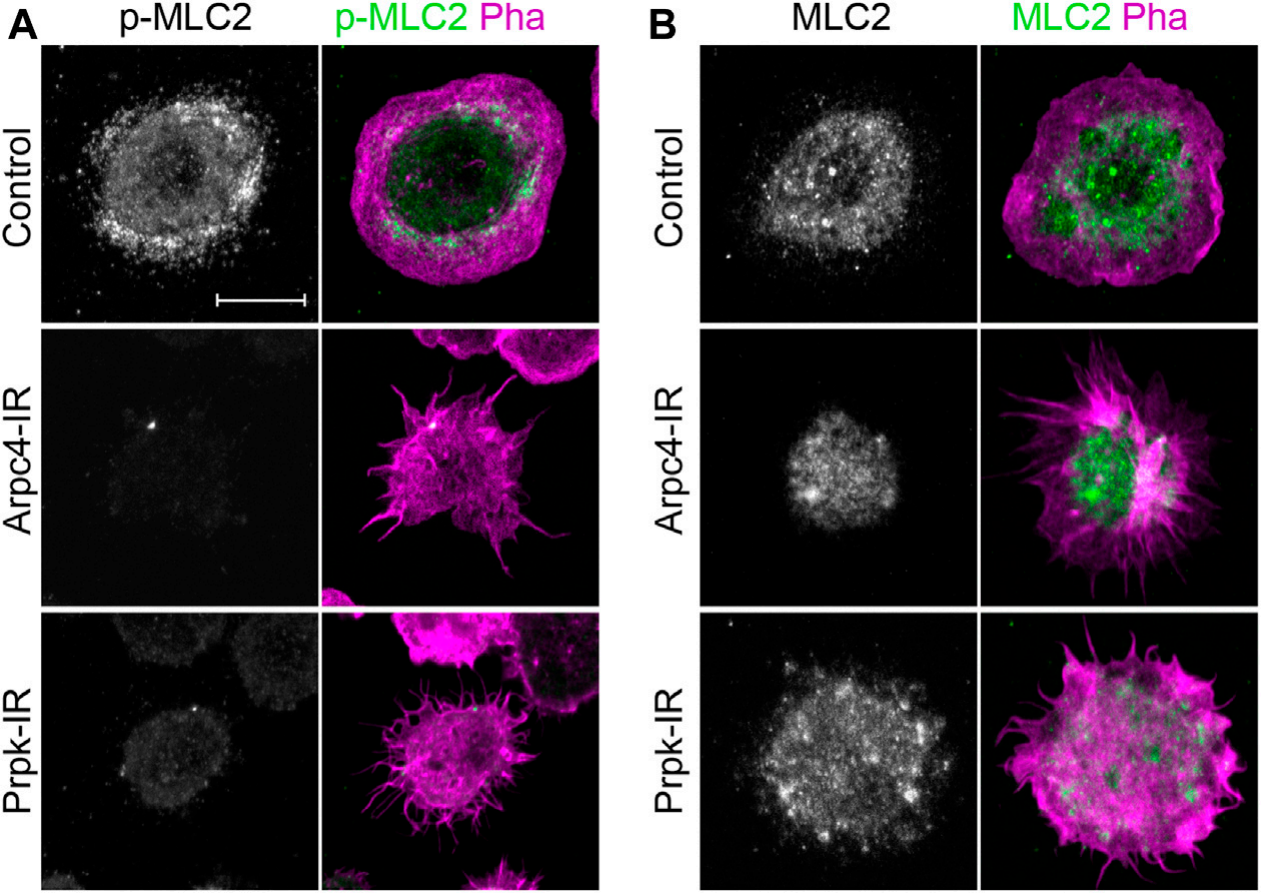
PRPK is required for proper activation of myosin-II. **(A)** Hemocyte immunofluorescence showing the localization of phospho-myosin light chain II (p-MLC-II, green) and stained for F-Actin (Pha, magenta). **(B)** Hemocyte immunofluorescence showing the localization of total myosin light chain II (MLC-II, green). Scale bars: 10 μm.

In addition to the formation of lamellipodia and filopodia, the actin-myosin network is also a key determinant for cell shape and migration (Burnette et al., 2011; Yang et al., 2012). In fibroblasts, the loss of function of the Ap2/3 complex alters the normal distribution of myosin-II (Suraneni et al., 2012, 2015). Similarly, in our culture conditions we observed a strong reduction in myosin light chain II phosphorylation (p-MLC-II) in hemocytes depleted of Prpk (Figure 7A), while the levels of total myosin II (MLC-II) were not visibly altered (Figure 7B). A possible explanation is that loss of lamellipodia and lamella redistribute Rac1 and Cdc42, which are responsible for activating MRCK and PAK, MLC-II kinases (Brzeska et al., 2004; Wilkinson et al., 2005; Zhao and Manser, 2015). MLC-II can also be phosphorylated by ROCK (Rok in *Drosophila*), which is activated by the RhoA GTPase in mammalian cells (Ueda et al., 2002) and by Rho1 in *Drosophila* (Jordan and Karess, 1997; Vasquez et al., 2014). However, we observed that expression of dominant negative versions of Rac1, Cdc42 or Rho1 did not generate a phenotype comparable to Prpk-IR (Supplementary Figure S1D). Furthermore, the expression of a dominant negative version of Rho1 in hemocytes generated multinucleated cells (Supplementary Figure S1D), likely due to its role in cytokinesis (Yoshida et al., 2009). Thus, we propose that the phenotype observed in hemocytes with low levels of Prpk is not due to a decrease in phosphorylated MLC-II levels *per se*.

## Discussion

Prpk is one of the few subunits of the TCTC complex that has been assigned a role outside the complex (Beenstock and Sicheri, 2021). It has been involved in functions such as: regulating the transcriptional activity of p53 (Abe et al., 2006), the regulation of axonal elongation (Villarroel-Campos et al., 2016) and the regulation of actin cytoskeleton dynamics in yeast and human podocytes (Ni and Snyder, 2001; Braun et al., 2017). Regarding the latter function, the first evidence connecting Prpk with actin dynamics, corresponds to the observation that Prpk mutants in yeast, besides displaying severe growth problems (Sartori et al., 2000), also exhibited a defective selection of the budding site (Ni and Snyder, 2001), a highly regulated and coordinated process where the actin cytoskeleton plays a fundamental role (Geymonat and Segal, 2017). More recently, human Prpk was shown to interact with the Arp2/3 complex in podocytes (Braun et al., 2017).

The Arp2/3 complex is widely accepted as being fundamental for hemocyte function; extensive evidence indicates that the Arp2/3 complex and its activators are indispensable elements for cell migration, phagocytosis, and immunological synapse formation (Machesky et al., 1999; Thrasher and Burns, 2010; Alekhina et al., 2017).

In this work we observed that the loss of function of Prpk mimicked the phenotypes obtained by silencing the Arp2/3 complex in both, hemocyte primary culture and in the *in-vivo* context of hemocytes random migration in the pupae. In the first scenario, Prpk knockdown hemocytes exhibited a phenotype characterized by the presence of numerous protrusions with very low dynamics and the lack of Fascin in those protrusions, very similar to what has already been described for the knockdown of SCAR/WAVE and depletions of Arp2/3 complex (Rogers et al., 2003; Biyasheva et al., 2004). In the *in-vivo* random migration scenario, we observed that the loss of function of both Prpk and the Arp2/3 complex significantly reduces the speed of migration, where Arp2/3 complex plays a fundamental role in integrin-dependent contexts such as haptotaxis (Wu et al., 2012; King et al., 2016; Rotty et al., 2017). In *Drosophila* it has been reported that random migration in the pupae mainly involves integrin-ECM interactions (Moreira et al., 2011, 2013; Ribeiro et al., 2014). Furthermore, knocking down Prpk diminished adhesion and the phagocytic capability of hemocytes, both processes also associated to Arp2/3 activity (King et al., 2016; Rotty et al., 2017), which directly affect the immune response and therefore the viability of infected animals. The molecular nature of these effects likely relies on the fact that Prpk co-localized in lamellipodia with the Arp2/3 complex (Arp2 and Arpc1 were observed in these work) and physically interacts with Arpc1. Overall, these finding suggest that Prpk interacts with the Arp2/3 complex and that Prpk silencing disrupts Arp2/3 subunit distribution and lamellipodia formation.

Then we analyzed the functional relationships of Prpk with different small GTPases (Rac1, Rho1, Cdc42 and Rab35) that influence Arp2/3 complex activity. Prpk physically interacts with Rab35 as it does in other models (Abe et al., 2006), suggesting the possible evolutionary conservation of this regulatory mechanism. Furthermore, Rab35 overexpression reversed the Prpk knockdown phenotype in primary cultures in a GTP/GDP dependent manner, since the dominant negative version of Rab35 was unable to reverse the phenotype, even slightly increasing the number of cells with protrusions. However, it is unlikely that Rab35 and Prpk act exclusively in the same pathway to regulate actin cytoskeleton, since a dominant negative form of Rab35 (or its knockdown) does not produce the morphological changes generated by Prpk-IR nor modify the phenotype induced by Prpk silencing (Zhang et al., 2009).

In pupal hemocytes Rab35 also rescued the Prpk knockdown phenotype. Conversely, overexpression of Rab35 did not rescue the phenotype of Arpc4 knockdown hemocytes, indicating that Arp2/3 is essential for the function of Rab35 in actin cytoskeleton organization. Interestingly, we reported that hemocytes overexpressing Rab35 exhibit both lamellipodia and filopodia, while hemocytes that co-expressed Rab35 and an Prpk-IR retain the ability to form filopodia but do not properly form lamellipodia. These results support the idea that Prpk is intimately related to the formation of lamellipodia and suggest that reversion of Prpk-IR phenotype elicited by Rab35 overexpression might be indirect, via its role in membrane recycling and the increase of Rac1 at the plasma membrane (Shim et al., 2010; Klinkert and Echard, 2016). Regarding this, we also determined that Rac1 overexpression was able to partially restore the actin dynamics that sustain cell shape and immune responses of Prpk knockdown hemocytes where the formation of Rac1-induced ruffles also requires Prpk.

Villarroel-Campos et al. showed that mice PRPK, depending on its interaction with MAP1B protein, negatively regulates axonal elongation by reducing the levels of Rab35 through the ubiquitin-proteasome pathway (Villarroel-Campos et al., 2016). These observations differ from ours, in that Prpk deficiencies did not resemble defects in Rab35 activity. Nonetheless, Villarroel-Campos et al. show as we do that PRPK participates in a process independent of the TCTC complex. Perhaps PRPK, similar to its *Drosophila* counterpart, possesses functions as constituent of the TCTC complex and independent to it. These functions might be based on the observation that yeast PRPK (Bud32/Tcs5p) acts as an ATPase in association with Kae1/Tcs3p, the catalytic subunit of TCTC complex, and as a kinase when dissociated from it (Perrochia et al., 2013). In addition to this, our results indicate that the ability of Prpk to regulate the dynamics of the actin cytoskeleton strongly depends on its kinase activity.

Another interestingly observation is the reduction in the MLC-II phosphorylation in Prpk knockdown hemocytes. We propose this phenotype is not necessarily related with Arp2/3 activity, since in other models, MLC-II activity was not affected under Arp2/3 complex deletion (Yang et al., 2012; Suraneni et al., 2015). Further research is required to clarify the connection between Prpk activity and these processes.

Finally, our findings describe a conserved role for Prpk in actin cytoskeleton dynamics, independent of its role in TCTC complex, translation and cell growth, which appears to be especially important for immune cells*.* Indeed, phagocytosis, hemocyte recruitment to wound sites, and random migratory parameters were all affected in Prpk deficient hemocytes. All these cellular behaviors depend on actin dynamics, which impact endocytosis, adhesion, and contractility. These alterations explain the inappropriate implementation of the immune response and the reduced survival of infected animals. In conclusion, Prpk, through the Arp2/3 complex function, supports actin dynamics to allow for the execution of cellular processes implicated in the correct implementation of the immune response by *Drosophila* hemocytes.

## Data Availability

The raw data supporting the conclusion of this article will be made available by the authors, without undue reservation.
