# Supplementary material for: p53 Related Protein Kinase is Required for Arp2/3-Dependent Actin Dynamics of Hemocytes in Drosophila melanogaster
Source: Front Cell Dev Biol. 2022 Jun 2;10:859105. doi: 10.3389/fcell.2022.859105 (PMC9201722; doi:10.3389/fcell.2022.859105)
Supplement: Supplementary file 4 [file DataSheet1.PDF]

SUPPLEMENTARY MATERIAL

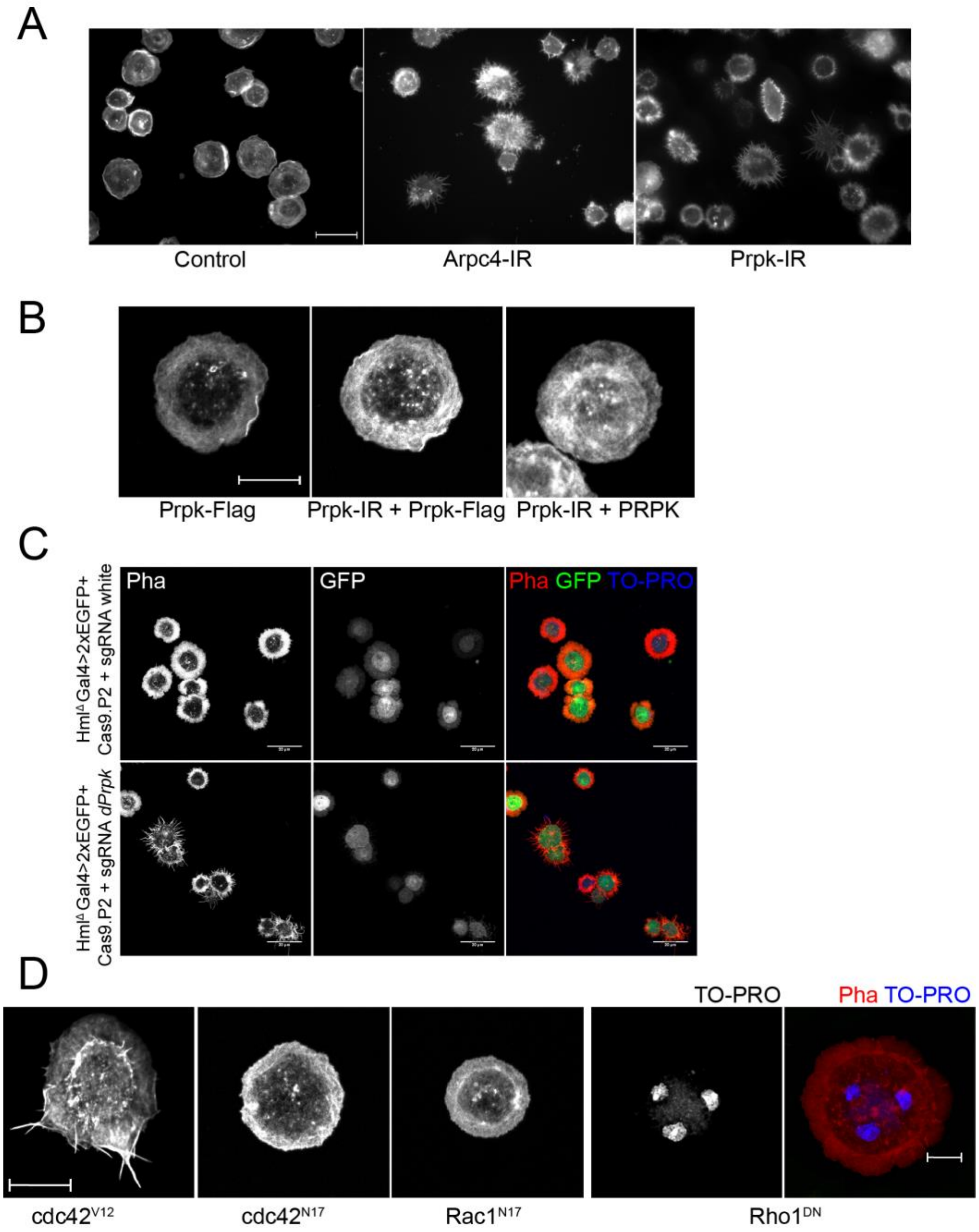

Figure S1. **Knockdown of Prpk and the lamellipodia actin nucleator Arp2/3 alter in similar manner hemocytes cell shape.** (**A)** Full-field images of F-actin stained with phalloidin in primary culture of hemocytes expressing: Driver Cg-Gal (Control), Arp2/3 p20 subunit RNAi (Arpc4-IR) and Prpk RNAi (Prpk-IR). Scale bar: 20 µm. (**B)** Confocal images of F-actin stained with phalloidin in primary culture of hemocytes expressing: Prpk-Flag, Prpk-IR + Prpk-Flag and Prpk-IR + PRPK. Scale bar= 10 µm. **(C)** Immunostaining of primary culture of hemocytes expressing sgRNA against *white* gene, sgRNA against *dPrpk*, Cas9.P2 and EGFP using the Hml^{Δ}-Gal4 driver. Scale bar= 20 µm. (**D)** Hemocytes expressing constitutively active form of Cdc42 ($Cdc42^{V12}$) and dominant negative forms of Cdc42 ($Cdc42^{N17}$), Rac1 ($Rac1^{N17}$) and Rho1 ($Rho1^{DN}$). F-actin (Pha) and nuclei (TO-PRO3) stains. Scale bar= 10 µm.

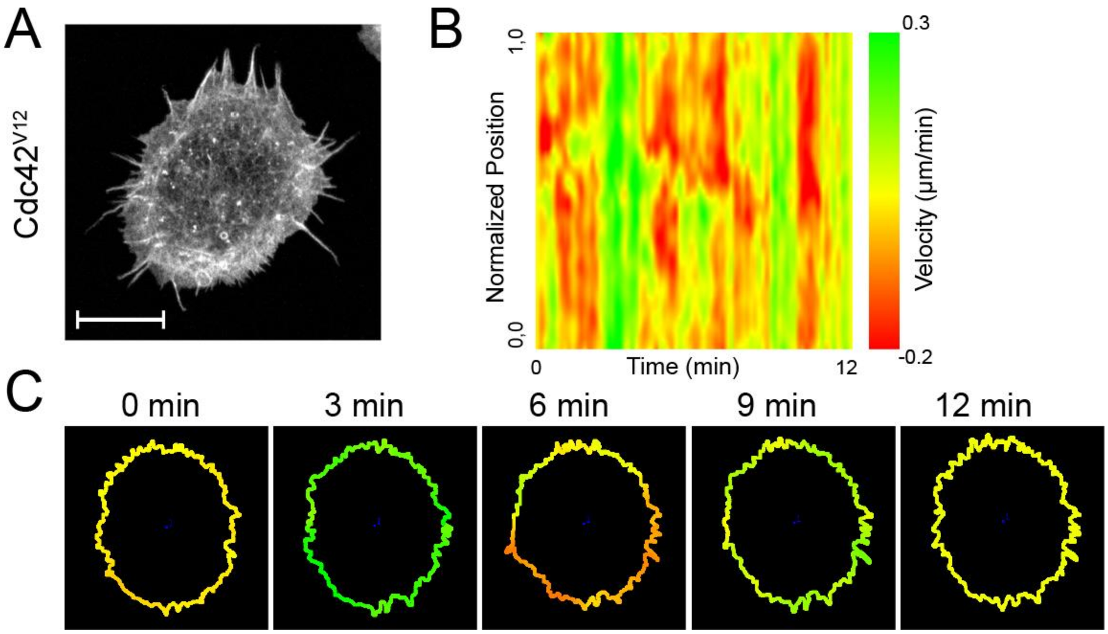

Figure S2. Expression of constitutively active form of Cdc42 (Cdc42^{V12}) induce filopodia formation. **(A)** Confocal image of F-actin stained with phalloidin of hemocyte expressing an active form of Cdc42 (Cdc42^{V12}). **(B)** Velocity map (Normalized position vs Time) generated from the variation in the fluorescence intensity in the periphery of the cell over time using the ADAPT tool. Regions that span the membrane are assigned a green color, while regions of retraction have a red color. The transition between green and red regions denotes the contractile activity of the plasma membrane. **(C)** Time-lapse of the actin cytoskeleton was performed co-expressing LifeAct-GFP with Cdc42^{V12}.

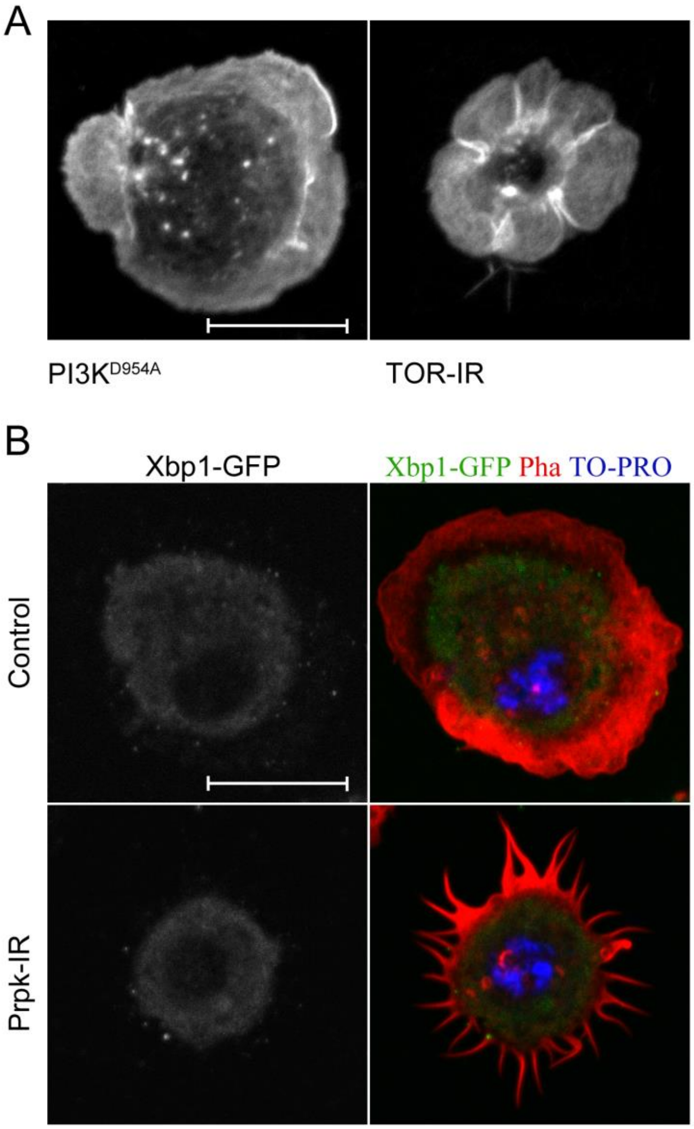

Figure S3. Prpk knockdown phenotype is not due to deficient cell growth or cellular stress due to faulty protein synthesis. **(A)** Confocal images of F-actin stained with phalloidin in primary culture of hemocytes expressing a dominant-negative form of PI3K ($PI3K^{D954A}$) or dsRNA against TOR (TOR-IR). **(B)** Hemocytes stained for F-actin with phalloidin (Pha, red), Xbp1-GFP (GFP, green) and nucleus with TO-PRO3 (DNA, blue). Scale bar: 10 μm.

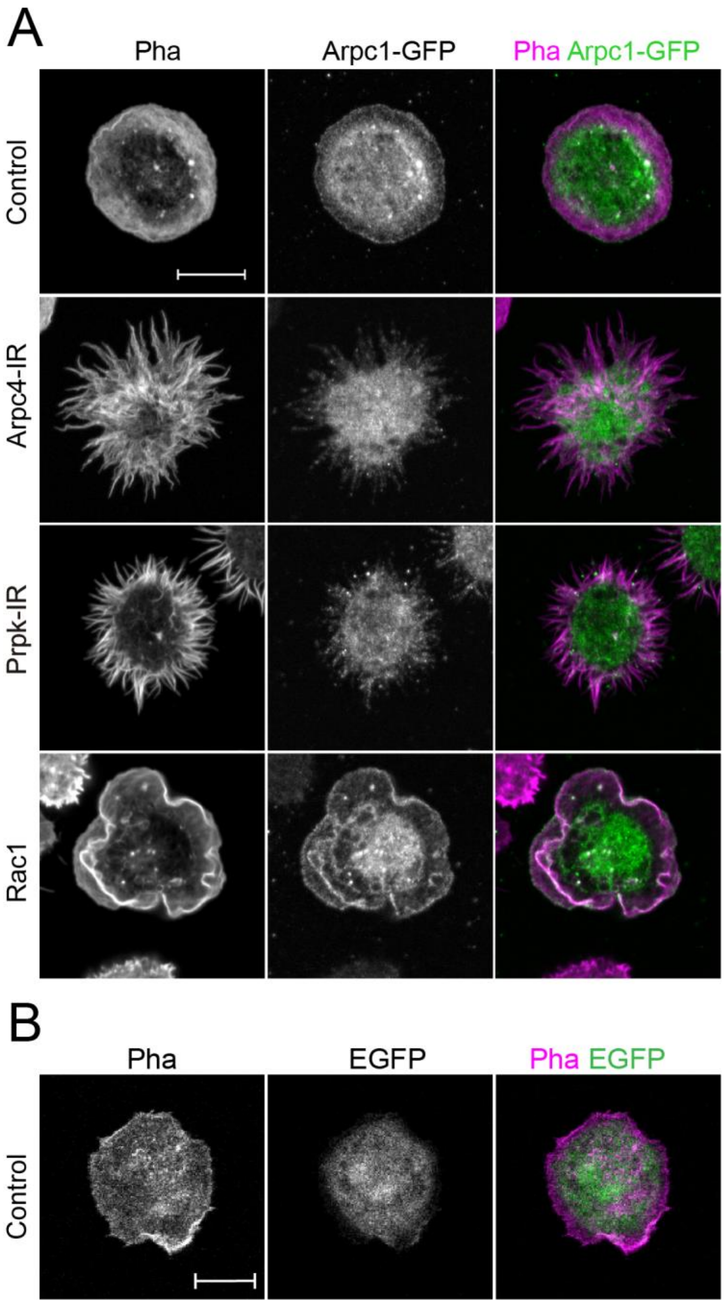

Figure S4. Prpk knockdown alters the distribution of Arpc1 (Arp2/3 subunit). (A) Immunostaining of primary culture of hemocytes expressing Arpc1-GFP stained for F-Actin (Pha, magenta) and GFP (green). Expressions: Arpc1-GFP (Control), Arpc4-IR + Arpc1-GFP (Arpc4-IR), Prpk-IR+ Arpc1-GFP (Prpk-IR) and Rac1 + Arcp1-GFP (Rac1). **(B)** Immunostaining of primary culture of hemocytes expressing EGFP stained for F-Actin (Pha) and EGFP. Scale bars: 10 µm.

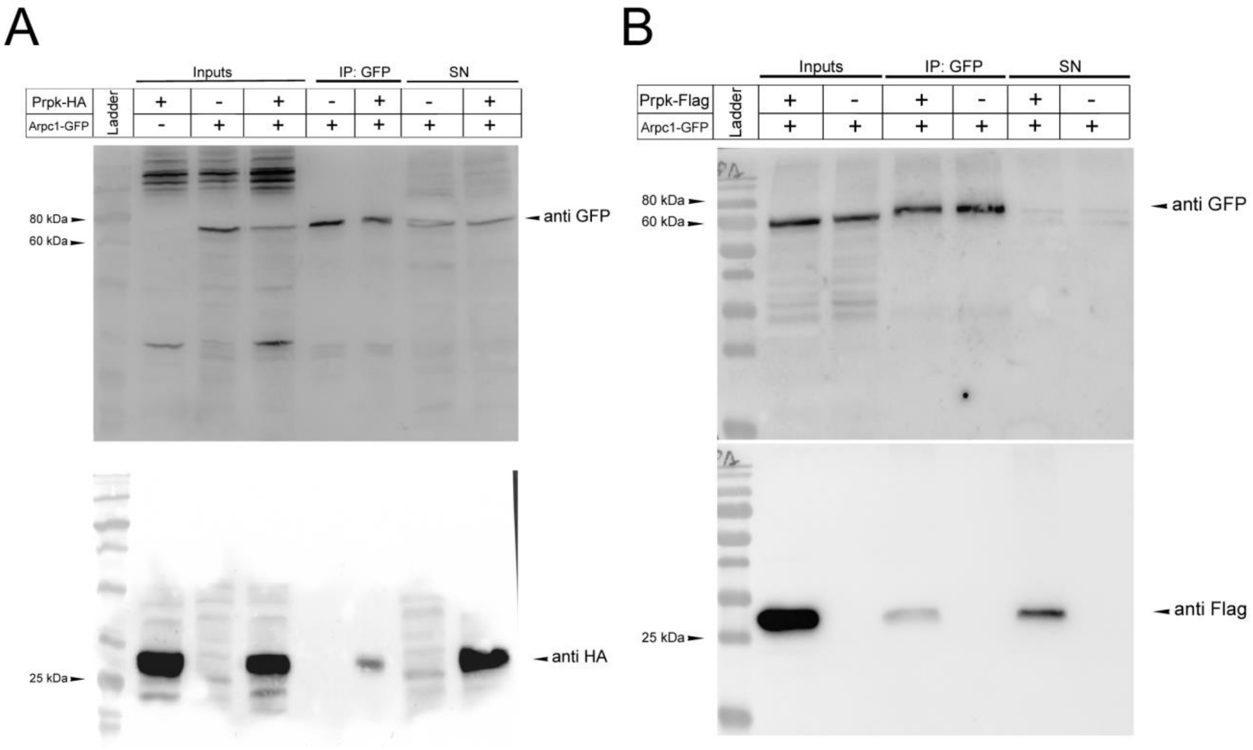

Figure S5. **Prpk interacts with Arpc1 (Arp2/3 complex subunit).** Co-immunoprecipitation of Arpc1-GFP and Prpk-HA (A) or Prpk-Flag (B). Both constructs were expressed using the Cg-gal4 driver. Immunoprecipitation were performed using GFP antibody and was revealed against GFP, Flag and HA. Inputs, Immunoprecipitation and Supernatants (SN) are detailed in each case. Each image corresponds to a representative blot, n=2. Construct expression was induced in hemocytes using the Cg-Gal4 driver.

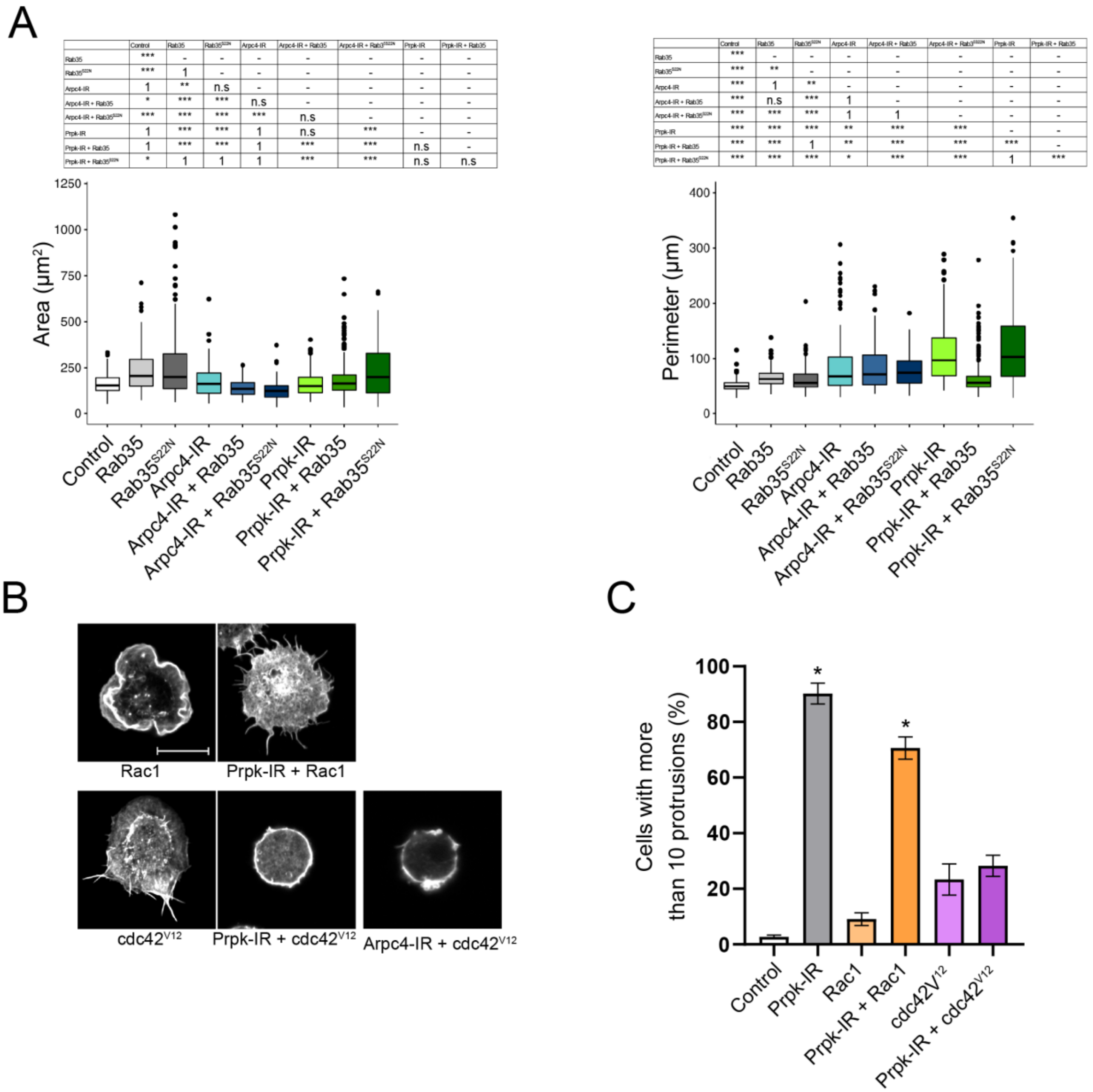

Figure S6. Rab35 and Rac1 partially rescue Prpk-IR phenotype in hemocytes. (A) Cell morphology quantification expressed like Area and perimeter for each condition detailed. Kruskal-Wallis test was used for data analysis, followed with pairwise comparisons using Wilcoxon rank sum test. The statistical differences are summarized in the inserted table. * $p< 0.05$, ** $p<0.01$, *** $p<0.001$; $n \geq 4$ (number of coverslips analyzed, quantified over 150 cells in each case) **(B)** Confocal images of F-actin stained with phalloidin in primary culture of hemocytes expressing: Rac1 and Cdc42^{V12} alone or co-expressed with Prpk RNAi (Prpk-IR + Rac1) (Prpk-IR + cdc42^{V12}). Also hemocytes expressing Arpc4 RNAi co-expressed with constitutive active form of Cdc42 (Arpc4-IR + Cdc42^{V12}). Constructs expression was induced using the Cg-Gal4 driver for each condition. Scale bar: 10 µm. **(C)** Quantification of the intensity of the Prpk-IR phenotypes. The co-expression of Rac1 partially rescues the Prpk knockdown phenotype. Data are shown as mean ± SD. * corresponds to $p<0.05$ between indicated conditions and Control. (Kruskal- Wallis test, n=4. 150 cells were counted for each experiment).

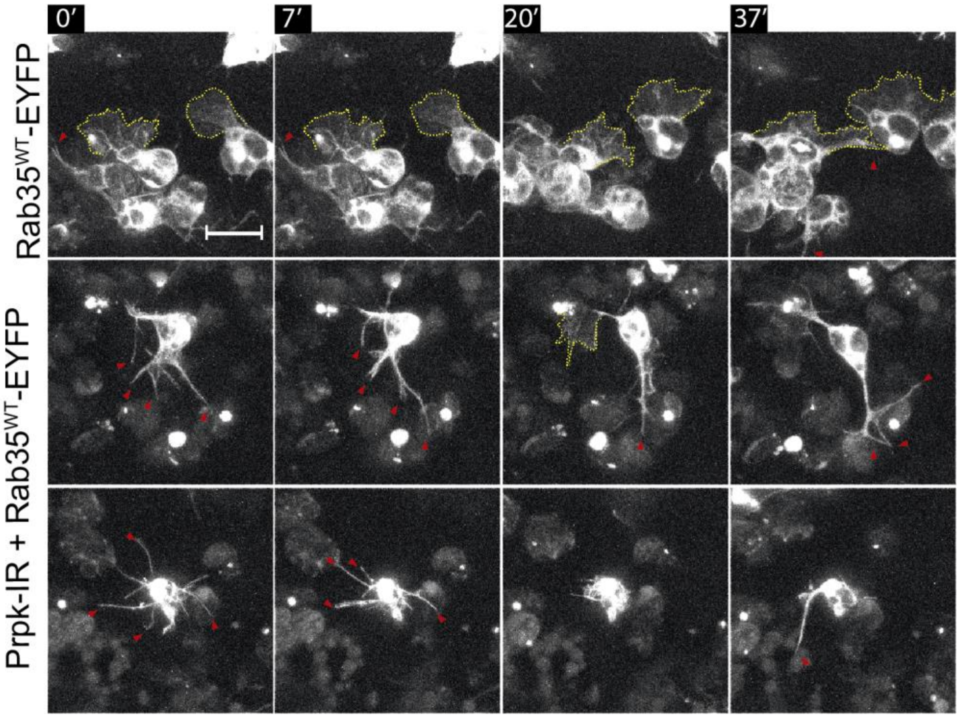

Figure S7. Co-expression of Prpk RNAi in pupal hemocytes overexpressing Rab35 alters the formation of lamellipodia and induces the formation of protrusions. Time-lapses of pupal hemocytes expressing Rab35 (Rab35^{WT}-EYFP) or co-expressing Prpk RNAi and Rab35 (Prpk-IR + Rab35^{WT}-EYFP) using the Hml$^{\Delta}$-Gal4 driver. Lamellipodia are outlined with a yellow dashed line, while filopodia are indicated with red arrowheads. Scale bar: 10 μm.

Supplementary Videos.

Supplementary Video 1. Actin cytoskeleton dynamics (LifeAct-GFP) of a representative Control hemocyte (Cg-Gal4> LifeAct-GFP). Films were made recording every 8 seconds for about 12 minutes using a Carl Zeiss LSM510 confocal microscope with a 100x objective for each condition. Individual hemocytes were recorded in the plane in which the largest membrane extension was observed. The span of the video is detailed in the upper left corner. Scale bar= 5 μm.

Supplementary Video 2. Live actin cytoskeleton imaging of a representative hemocyte expressing an RNAi against Arp2/3 p20 subunit (Arpc4-IR) (Cg-Gal4> LifeAct-GFP; Arpc4-IR). The video was made similar to Supplementary Video 1. Individual hemocytes were recorded in the plane in which the largest membrane extension was observed. The span of the video is detailed in the upper left corner. Scale bar= 5 μm.

Supplementary Video 3. Live actin cytoskeleton imaging of a representative hemocyte expressing an RNAi against Prpk (Prpk-IR) (Cg-Gal4> LifeAct-GFP; Prpk-IR) using LifeAct-GFP. The video was made similar to previous videos. Individual hemocytes were recorded in the plane in which the largest membrane extension was observed. The span of the video is detailed in the upper left corner. Scale bar= 5 μm.

Supplementary Video 4. Live actin cytoskeleton imaging of a representative hemocyte overexpressing the constitutive active form of Cdc42 ($Cdc42^{V12}$) (Cg-Gal4> LifeAct-GFP; $Cdc42^{V12}$) using LifeAct-GFP. The video was made similar to previous videos. The span of the video is detailed in the upper left corner. Scale bar= 5 μm.

Supplementary Video 5. Time-lapse of Control pupal hemocytes of 15-20 hours after puparium formation (apf) expressing GFP specifically in hemocytes with the Hml-Gal4 driver (Hml-Gal4> 2xEGFP). Videos are Z-projections of 15 slices of 1 μm each acquired every 2 minutes for 30 minutes. Scale bar= 50 μm.

Supplementary Video 6. Time-lapse of pupal hemocytes overexpressing $Rab35^{WT}$-YFP of 15-20 hours after puparium formation (apf) expressing GFP specifically in hemocytes with the Hml-Gal4 driver (Hml-Gal4> 2xEGFP). Videos are Z-projections of 15 slices of 1 μm each acquired every 2 minutes for 30 minutes. Scale bar= 50 μm.

Supplementary Video 7. Time-lapse of Prpk-IR pupal hemocytes of 15-20 hours after puparium formation (apf) expressing Prpk-IR and GFP in hemocytes with the Hml-Gal4 driver (Hml-Gal4> 2xEGFP; Prpk-IR). Videos are Z-projections of 15 slices of 1 μm each acquired every 2 minutes for 30 minutes using a Carl Zeiss LSM510 confocal microscope with a 40x objective. Scale bar= 50 μm.

Supplementary Video 8. Time-lapse of pupal hemocytes co-overexpressing Prpk-IR and $Rab35^{WT}$-YFP of 15-20 apf expressing GFP in hemocytes with the Hml-Gal4 driver (Hml-Gal4> 2xEGFP). Videos are Z-projections of 15 slices of 1 μm each acquired every 2 minutes for 30 minutes. Scale bar= 50 μm.

Supplementary Video 9. Time-lapse of Arpc4-IR pupal hemocytes of 15-20 hours after puparium formation (apf) expressing an RNAi against Prpk (Prpk-IR) and GFP specifically in hemocytes with the Hml-Gal4 driver (Hml-Gal4> 2xEGFP; Prpk-IR). Videos are Z-projections of 15 slices of 1 μm each acquired every 2 minutes for 30 minutes. Scale bar= 50 μm.
